# Increasing Fatty Acid Oxidation Remodels the Hypothalamic Neurometabolome to Mitigate Stress and Inflammation

**DOI:** 10.1371/journal.pone.0115642

**Published:** 2014-12-26

**Authors:** Joseph W. McFadden, Susan Aja, Qun Li, Veera V. R. Bandaru, Eun-Kyoung Kim, Norman J. Haughey, Francis P. Kuhajda, Gabriele V. Ronnett

**Affiliations:** 1 Center for Metabolism and Obesity Research, The Johns Hopkins University School of Medicine, Baltimore, Maryland, United States of America; 2 Department of Neuroscience, The Johns Hopkins University School of Medicine, Baltimore, Maryland, United States of America; 3 Department of Neurology, The Johns Hopkins University School of Medicine, Baltimore, Maryland, United States of America; 4 Department of Pathology, The Johns Hopkins University School of Medicine, Baltimore, Maryland, United States of America; 5 Department of Biological Chemistry, The Johns Hopkins University School of Medicine, Baltimore, Maryland, United States of America; 6 Department of Brain Science, Daegu Gyeongbuk Institute of Science and Technology, Daegu, South Korea; Nihon University School of Medicine, Japan

## Abstract

Modification of hypothalamic fatty acid (FA) metabolism can improve energy homeostasis and prevent hyperphagia and excessive weight gain in diet-induced obesity (DIO) from a diet high in saturated fatty acids. We have shown previously that C75, a stimulator of carnitine palmitoyl transferase-1 (CPT-1) and fatty acid oxidation (FAOx), exerts at least some of its hypophagic effects via neuronal mechanisms in the hypothalamus. In the present work, we characterized the effects of C75 and another anorexigenic compound, the glycerol-3-phosphate acyltransferase (GPAT) inhibitor FSG67, on FA metabolism, metabolomics profiles, and metabolic stress responses in cultured hypothalamic neurons and hypothalamic neuronal cell lines during lipid excess with palmitate. Both compounds enhanced palmitate oxidation, increased ATP, and inactivated AMP-activated protein kinase (AMPK) in hypothalamic neurons in vitro. Lipidomics and untargeted metabolomics revealed that enhanced catabolism of FA decreased palmitate availability and prevented the production of fatty acylglycerols, ceramides, and cholesterol esters, lipids that are associated with lipotoxicity-provoked metabolic stress. This improved metabolic signature was accompanied by increased levels of reactive oxygen species (ROS), and yet favorable changes in oxidative stress, overt ER stress, and inflammation. We propose that enhancing FAOx in hypothalamic neurons exposed to excess lipids promotes metabolic remodeling that reduces local inflammatory and cell stress responses. This shift would restore mitochondrial function such that increased FAOx can produce hypothalamic neuronal ATP and lead to decreased food intake and body weight to improve systemic metabolism.

## Introduction

Overnutrition-induced metabolic dysfunction is a severe health concern in both industrialized and developing countries. Sustained exposure to excess dietary fatty acids (FA) causes lipid accumulation in non-adipose tissues with limited storage capacity. This lipotoxicity causes cellular stresses and inflammation that lead to cell damage [1], and in peripheral tissues contributes to insulin resistance and metabolic syndrome [2], [3]. The hypothalamus is similarly vulnerable to lipotoxicity. The hypothalamus senses availability of nutrients, including fat, to control food intake and regulate energy balance [4], [5]. However, elevated saturated FA is sufficient to induce lipotoxic stress in the hypothalamus and attenuate responses to insulin and leptin negative feedback, contributing to dietary-induced obesity (DIO) and attendant metabolic dysfunction [6]–[8].

Long-chain FA signals nutrient surplus in hypothalamus [5], and modulating FA catabolic and anabolic processing can alter feeding behavior [9]. In this regard, we previously targeted key enzymes of FA metabolism: fatty acid synthase (FAS), carnitine palmitoyltransferase-1 (CPT-1), and glycerol-3-phosphate acyltransferases (GPATs). FAS catalyzes ATP- and NADH-dependent palmitate synthesis [10]. CPT-1 catalyzes long-chain FA translocation into mitochondria for β-oxidation [11]. C75 is a FAS inhibitor and CPT-1 stimulator [12] that decreases expression of orexigenic neuropeptides agouti-related protein (AgRP) and neuropeptide Y (NPY) in the arcuate nucleus [13], [14] to decrease food intake and increase energy expenditure [15]. These effects of C75 rely less on FAS inhibition and more on CPT-1 stimulation and FAOx [12], [16]; thus, i.c.v. injection of C89b, a CPT-1 stimulator that does not affect FAS activity, elicits persistent hypophagia and weight loss [17]. GPATs have emerged as another target for appetite suppression and weight loss. GPATs catalyze the first esterification step for acylglycerol and phospholipid syntheses [18]. We showed that the GPAT inhibitor FSG67 [19] given i.p. or i.c.v. elicits hypophagia and weight loss [20]. Mechanisms by which pharmacologic modification of hypothalamic FA metabolism produces these effects are being elucidated.

Fluctuating AMP∶ATP ratio may serve as a signal common to both hypothalamic nutrient sensing and appetite control [21], by altering the activity of AMP-activated protein kinase (AMPK), an energy-sensor that regulates both intracellular and body energy balance [22]. With high AMP∶ATP ratio, AMPK is phosphorylated and activated (pAMPK) to preserve and produce ATP by multiple means, including fat catabolism. Food restriction decreases hypothalamic ATP [21], and whereas food restriction and orexigenic signals increase hypothalamic pAMPK to increase ingestion, nutrients and other negative feedback signals decrease hypothalamic pAMPK and food intake [23]. We showed that the CPT-1 stimulator and FAS inhibitor C75 increases hypothalamic neuronal ATP and decreases pAMPK to curtail feeding [12], [14]. How altering FA flux might affect other aspects of hypothalamic neuronal metabolism to contribute to these effects needs to be explored.

While oxidative metabolism produces ATP, it also generates reactive oxygen species (ROS). Sustained high levels of ROS lead to oxidative stress and impaired mitochondrial function and ATP production [24]. Increased ROS can also cause unfolded or misfolded proteins to accumulate in the endoplasmic reticulum (ER). This ER stress initiates the unfolded protein response (UPR) [25] that reduces protein translation generally, but upregulates expression of transcription factors X-box binding protein-1 (XBP1) and activating transcriptional factor (ATF) 4 and ATF6, to increase ER chaperone and degradation machinery that optimize protein folding. Thus, overnutrition induces hypothalamic ER stress, leading to insulin and leptin resistance and obesity [6]. Excess palmitate induces ER stress and apoptosis in the mHypoE-44 hypothalamic cell line [26], while CNS administration of an ER stress inducer inhibits the hypophagic effects of leptin and insulin [27]. However, the relationships between FA metabolism and hypothalamic stress during nutrient excess have not been fully defined, and may be critical to our understanding of mechanisms that could be targeted to remediate metabolic imbalance in obesity.

Overnutrition also leads to chronic inflammation, characterized by elevated interleukin (IL) 6, IL1β, and tumor-necrosis factor-α (TNFα). Inflammation, potentially mediated by ER stress [2], is involved in development and pathogenesis of insulin resistance and metabolic syndrome [3]. The hypothalamus is susceptible to inflammation from saturated FA [7], [8]. Mice with hypothalamic FAS deletion are protected from DIO and inflammation [28]; therefore, controlling hypothalamic FA metabolism might prevent neuronal inflammation and its contribution to DIO.

Here, we used compounds that alter FA metabolism, and are known to decrease food intake and body weight at least in part by altering hypothalamic neuronal energy status [14], [29], [30]. We examined their effects on the metabolome, oxidative and ER stresses, and inflammation responses in hypothalamic neurons and validated hypothalamic neuronal cell lines in culture. We show that CPT-1 stimulation or GPAT inhibition, even in the presence of excess saturated FA, modifies energy metabolism and gene transcription in ways that increase ATP levels in neurons in vitro. Our results indicate that increasing FAOx in hypothalamic neurons modifies their metabolome to prevent oxidative and ER stress and inflammation.

## Results

### FA catabolism increases ATP in hypothalamic neurons

**Figure 1:**
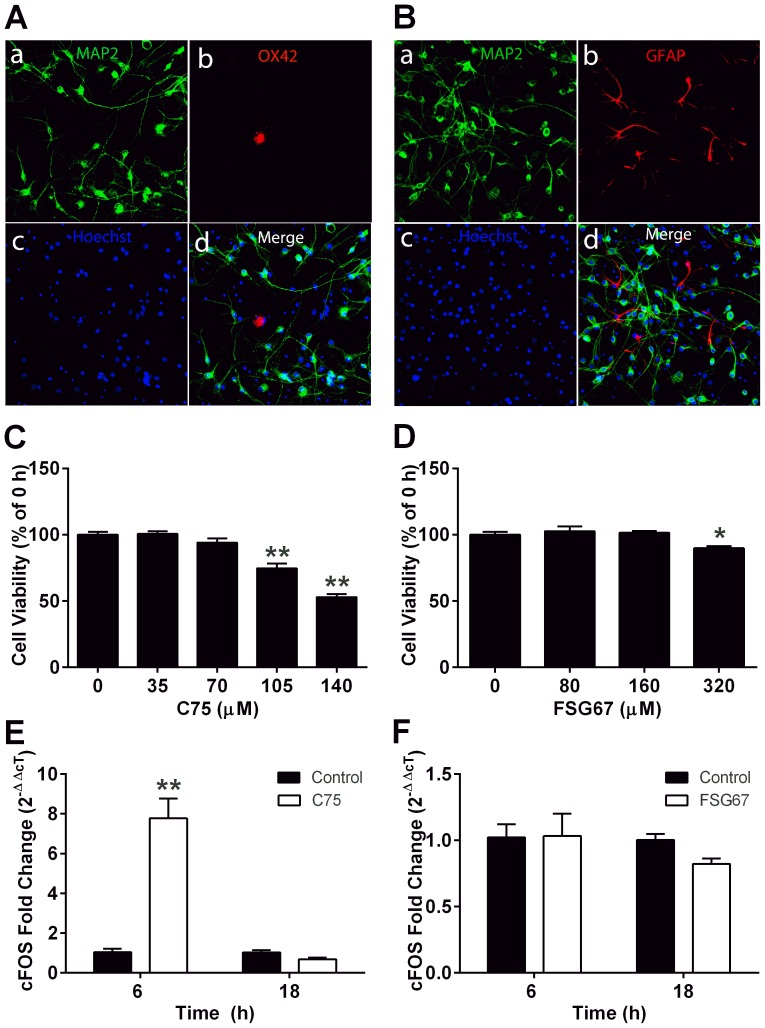
Cell viability and cFOS mRNA expression in PHN cultures after incubation with C75 or FSG67. PHN immunostains showed (A) 85% MAP2-positive neurons, 0.41% OX42-positive microglia, and (B) 4.8% GFAP-positive astrocytes. Hoechst 33342 stained nuclear DNA. Treating PHN with (C) 70 µM C75 or (D) 160 µM FSG67 for 24 h did not alter cell viability. Acute treatment of PHN with (E) C75 increased cFOS mRNA, but (F) FSG67 did not alter cFOS mRNA expression. Cell viability data were from two experiments, four to six replicates each. cFOS data were from two independent experiments, each with two or three replicates. For all data: **, *p*<0.01; *, *p*<0.05. Data are represented as means ± SEM.

We utilized primary hypothalamic neurons (PHN), maintained in conditions with glucose and oxygen at levels that are physiological for brain [31], [32], to measure the effects of C75 or FSG67 on FA metabolism, ATP, and AMPK phosphorylation. Cultures were 85% neurons, 0.4% microglia and 4.8% astrocytes (Fig. 1A, 1B). In additional studies, we used validated hypothalamic neuronal lines to confirm that effects observed in PHN cultures were attributable to neurons. Treatments of PHN for 24 h with C75 up to 70 µM, or with FSG67 up to 160 µM, did not alter cell viability (Fig. 1C, 1D), similar to results in rat R7HN and mouse N38HN (data not shown), immortalized hypothalamic cell lines that express NPY and AgRP. We measured cFOS mRNA in PHN to assess this index of neuronal activation. C75 for 6 h increased cFOS expression in PHN (Fig. 1E), as it did in hypothalamic neurons in vivo [30], while FSG67 did not (Fig. 1F).

**Figure 2:**
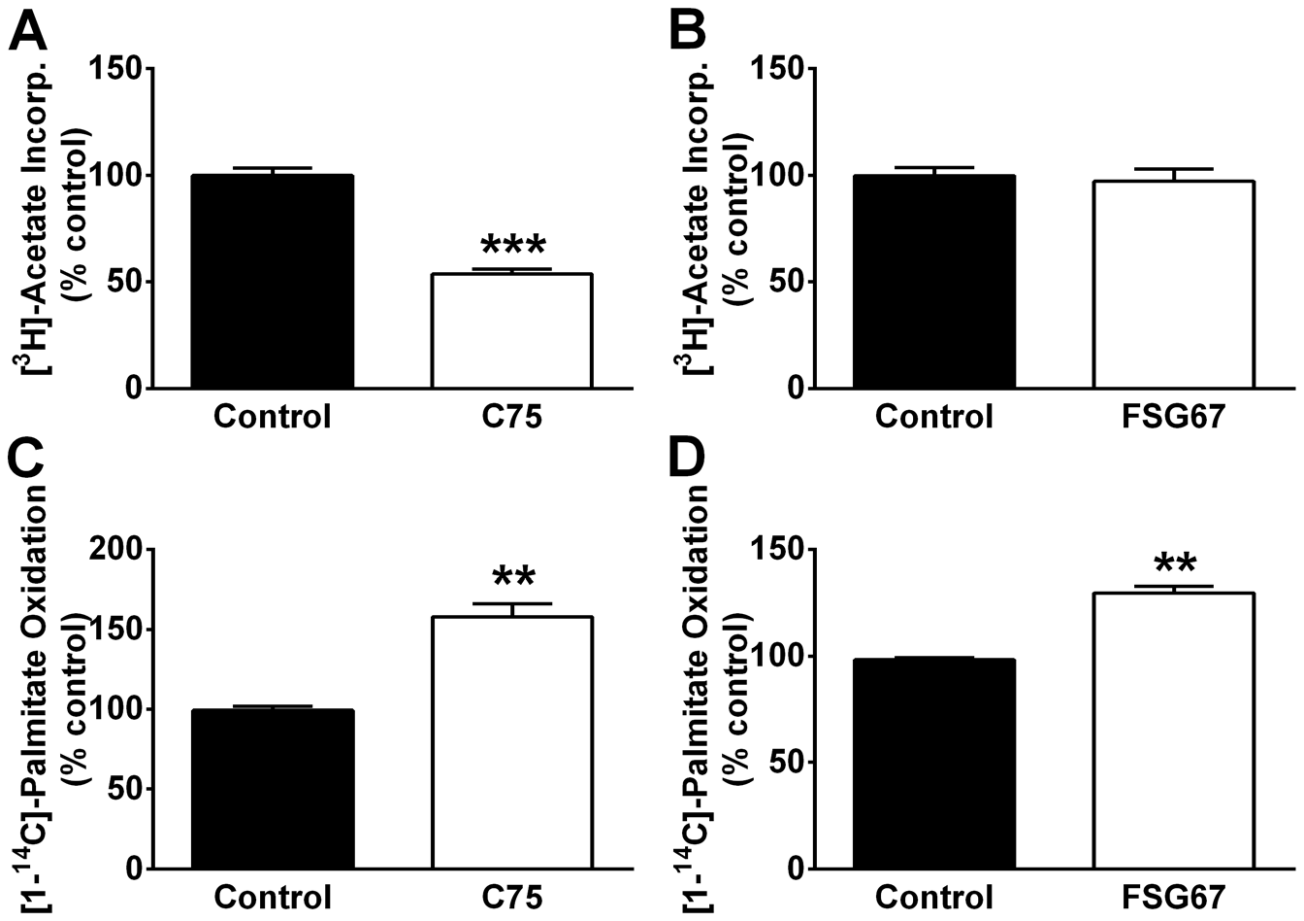
C75 and FSG67 increase FA catabolism. C75 for 2 h inhibited FA synthesis in PHN (A). (B) FSG67 for 2 h did not affect FA synthesis. (C) Palmitate oxidation in PHN was increased after 4 h with C75 or (D) FSG67. Data are represented as means ± SEM, from two independent experiments, each with two or three replicates. For all data: ***, *p*<0.001; **, *p*<0.01.

C75 decreased acetate incorporation into lipids in PHN, and thus inhibited FA synthesis (Fig. 2A), as seen previously in cortical neuron cultures [12]. FSG67 did not affect acetate incorporation (Fig. 2B), as anticipated due to its design as a GPAT inhibitor. C75 increased palmitate oxidation in PHN (Fig. 2C) and N38HN (S1A Fig.), as it did in other neuronal cultures [12]. FSG67 also increased palmitate oxidation in PHN (Fig. 2D). FSG67 may enhance FAOx by decreasing esterification, thus making FA available to CPT-1 for transport into mitochondria for oxidation.

**Figure 3:**
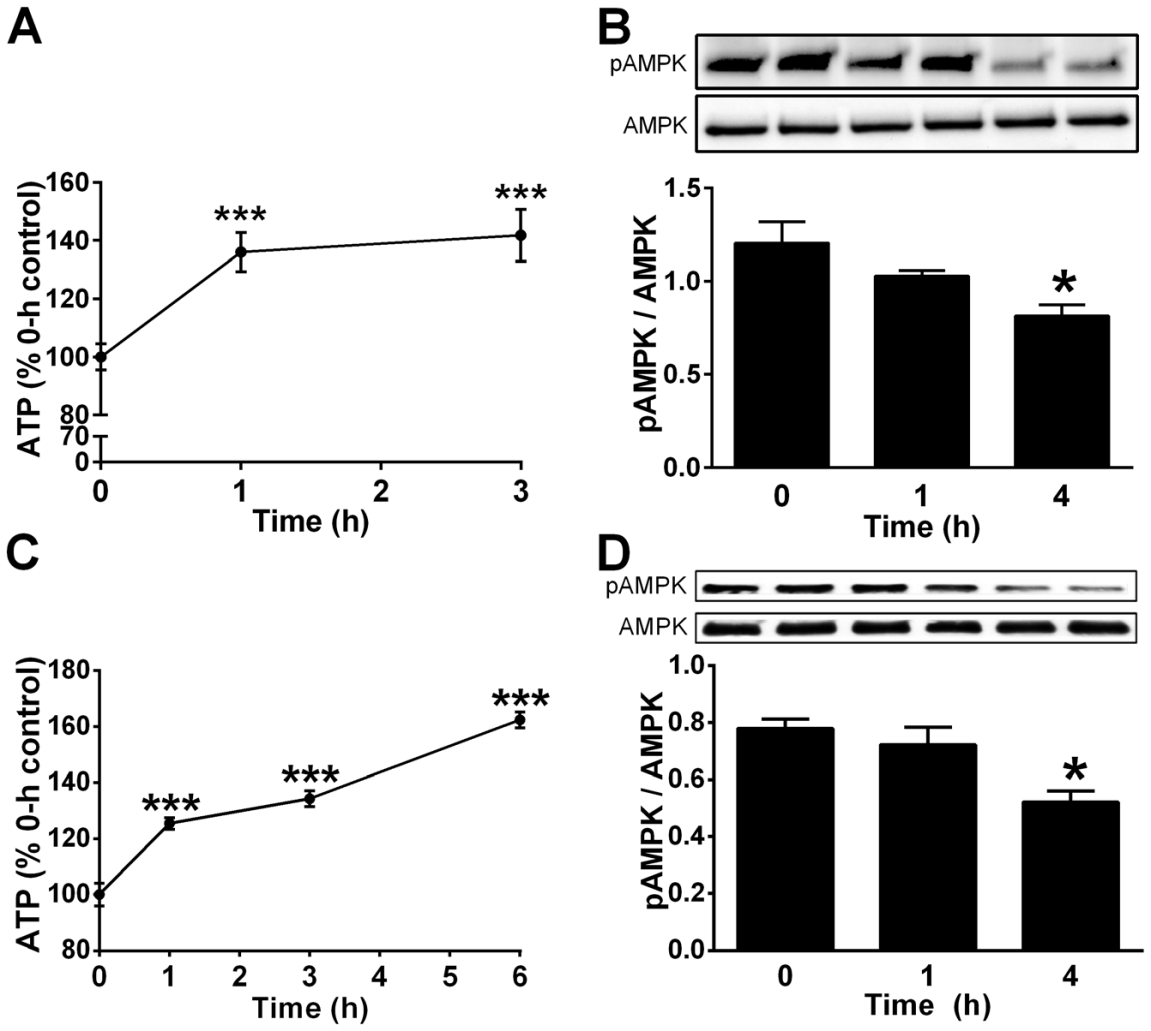
C75 and FSG67 increase ATP, and inactivate AMPK. (A) C75 increased ATP in PHN, and inactivated AMPK (B). In PHN, FSG67 (C) increased ATP and (D) inactivated AMPK. ATP data were from two experiments, four to six replicates each. AMPK data were from two independent experiments, each with two or three replicates. Data are represented as means ± SEM. For all data: ***, *p*<0.001; **; *, *p*<0.05.

We investigated whether FSG67, like C75, could increase ATP in hypothalamic neurons. C75 increased ATP in PHN (Fig. 3A), and decreased active pAMPK (Fig. 3B), as shown previously [14]. FSG67 likewise increased ATP and decreased pAMPK (Fig. 3C, 3D). In N38HN, both compounds produced biphasic responses in ATP levels and pAMPK phosphorylation, as seen with C75 in neurons [12]; ATP decreased and then increased (S1B Fig.), with reciprocal changes in pAMPK (S1C Fig.).

### C75 and FSG67 alter transcription of CPT-1 and GPAT isoforms in PHN

**Figure 4:**
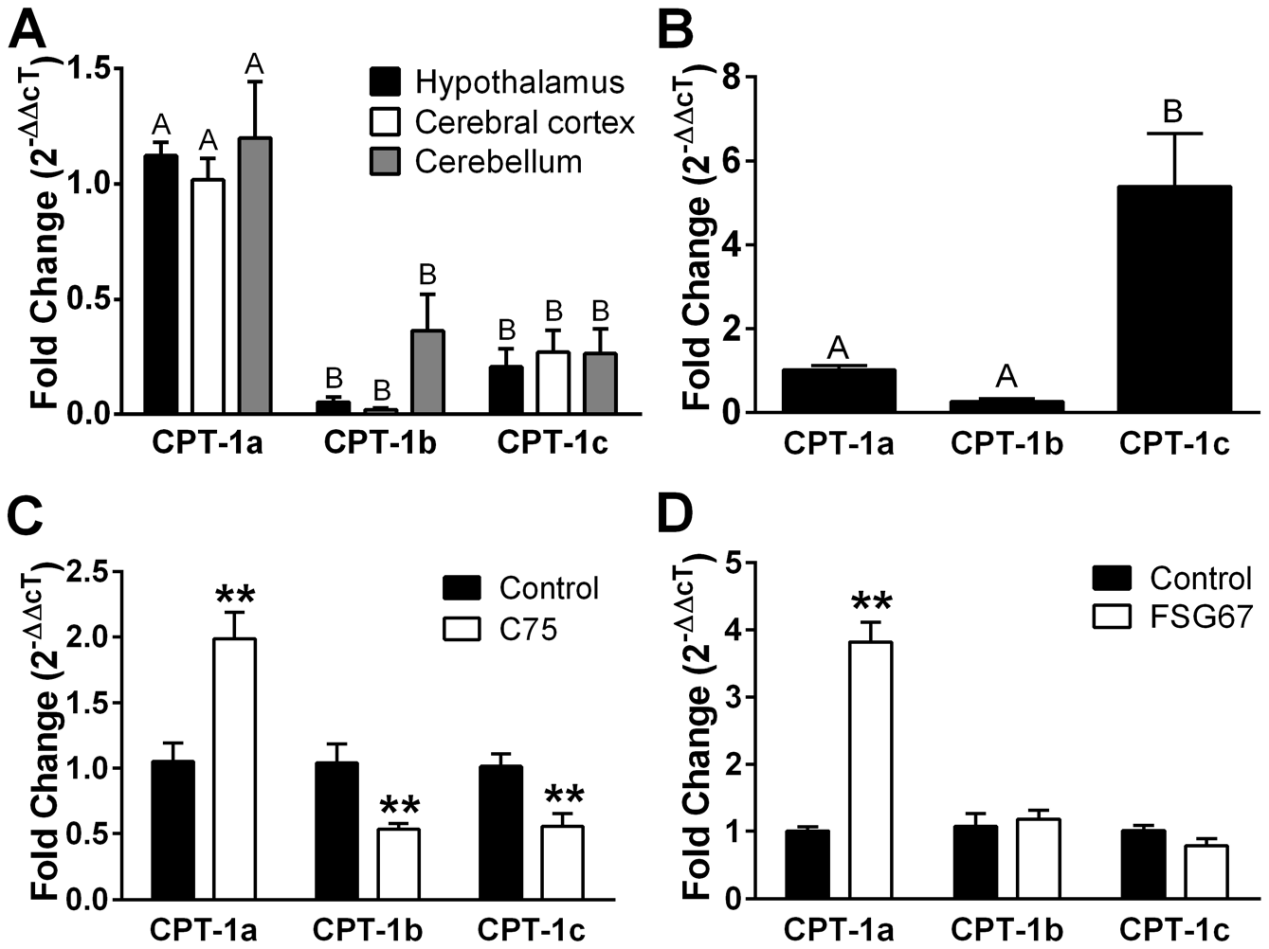
C75 and FSG67 modify transcription of CPT-1 isoforms in PHN. (A) Brain tissues from rat primarily express CPT-1a; however, (B) the CPT-1c isoform is predominant in PHN cultures. In PHN, exposure for 18 h to (C) C75 or (D) FSG67 increased CPT-1a expression. For tissue mRNA data, *n* = 6/tissue. Data are represented as means ± SEM. CPT-1a mRNA levels were baseline for comparisons within tissue and within isoform. Treatment differences are signified by differing superscripts within tissue, *p*<0.01. For PHN data: **, *p*<0.01.

CPT-1 is the rate-limiting step for β-oxidation of long-chain FA, so we measured the expression of isoforms in adult rat brain and in PHN. CPT-1a and CPT-1b catalyze acyl transfer from CoA to carnitine for transport across the outer mitochondrial membrane. CPT-1c is located on neuronal ER, and has lower catalytic efficiency than the other isoforms [33], but appears to have a role in energy balance [34]. CPT-1a was predominant in adult rat hypothalamus, cerebral cortex, and cerebellum (Fig. 4A). Mitochondrial CPT-1a is the most widely expressed isoform in brain, and CPT-1a expression is equally abundant in cultured neurons versus astrocytes [35]. CPT-1c was most prevalent in PHN (Fig. 4B), confirming the high degree of neuronal enrichment in the cultures. Both C75 and FSG67 increased CPT-1a expression in PHN (Fig. 4C, 4D), an effect that would further support their effect to increase palmitate oxidation. C75 also decreased CPT-1c expression (Fig. 4C), which may reflect a shift of FA flux away from the ER and toward mitochondrial uptake.

**Figure 5:**
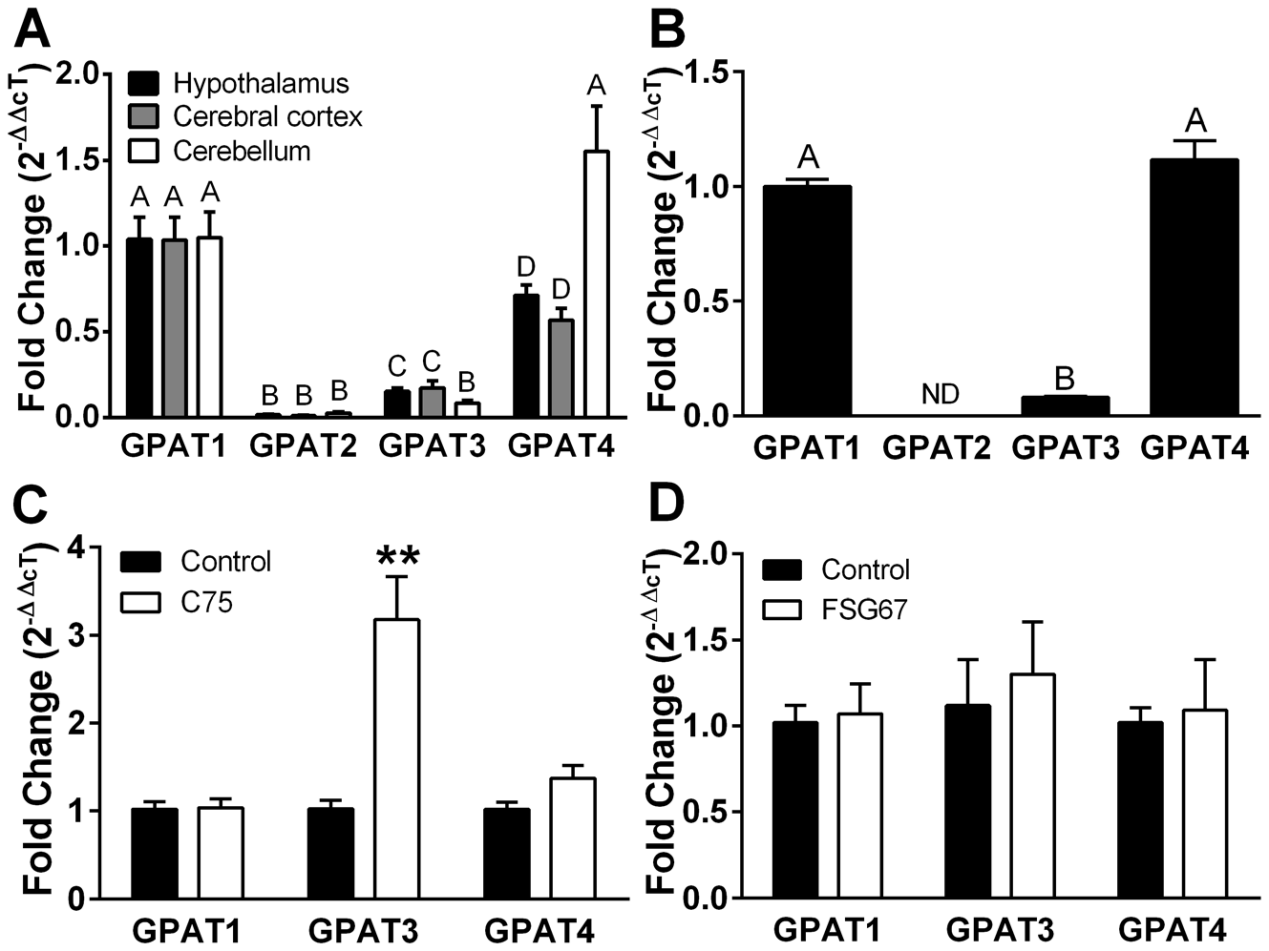
C75 and FSG67 modify transcription of GPAT isoforms in PHN. (A) Brain tissues and (B) PHN cultures express mainly GPAT1 and GPAT4. GPAT2 was barely or not detected (ND). In PHN, exposure for 18 h to (C) C75 increased GPAT3 mRNA. (D) FSG67 did not alter expression of GPAT isoforms. For tissue mRNA data, *n* = 6/tissue. Data are represented as means ± SEM. GPAT1 mRNA levels were baseline for comparisons within tissue and within isoform. Treatment differences are signified by differing superscripts within tissue, *p*<0.01.

Because GPAT inhibition altered FA metabolism in PHN, we also measured expression levels of the four GPAT isoforms in rat brain and PHN. GPAT1 and GPAT2 reside in the outer mitochondrial membrane, whereas microsomal GPAT3 and GPAT4 localize to ER [36]. GPAT1 and GPAT4 were predominant in rat hypothalamus, cortex, and cerebellum (Fig. 5A), as well as in PHN (Fig. 5B), consistent with adult mouse brain (S2A Fig.) and N38HN (S2B Fig.). Chronic C75 increased PHN expression of GPAT3 (Fig. 5C); this may ensure membrane integrity during the increased β-oxidation and inhibited FA synthesis. FSG67 did not alter GPAT expression (Fig. 5D), indicating that the decreased GPAT activity seen with FSG67 [19], [20] is not due to decreased gene expression, but rather to direct inhibition of the enzyme.

Sterol regulatory element-binding protein-1c (SREBP1c) controls gene transcription for lipogenic enzymes such as acetyl-CoA carboxylase (ACC), FAS, and GPAT. We measured SREBP1c and FAS expression after C75 or FSG67. C75 decreased SREBP1c and FAS transcription (S3A Fig.), as seen in vivo [29]. Decreased FAS expression would help to decrease flux through the FA synthetic pathway and preserve ATP. FSG67 did not alter expression of SREBP1c or FAS (S3B Fig.), supporting the hypothesis that the increased ATP results from increased FAOx rather than decreased ATP usage to synthesize FA.

### Enhanced FAOx increases ROS, but not oxidative stress

**Figure 6:**
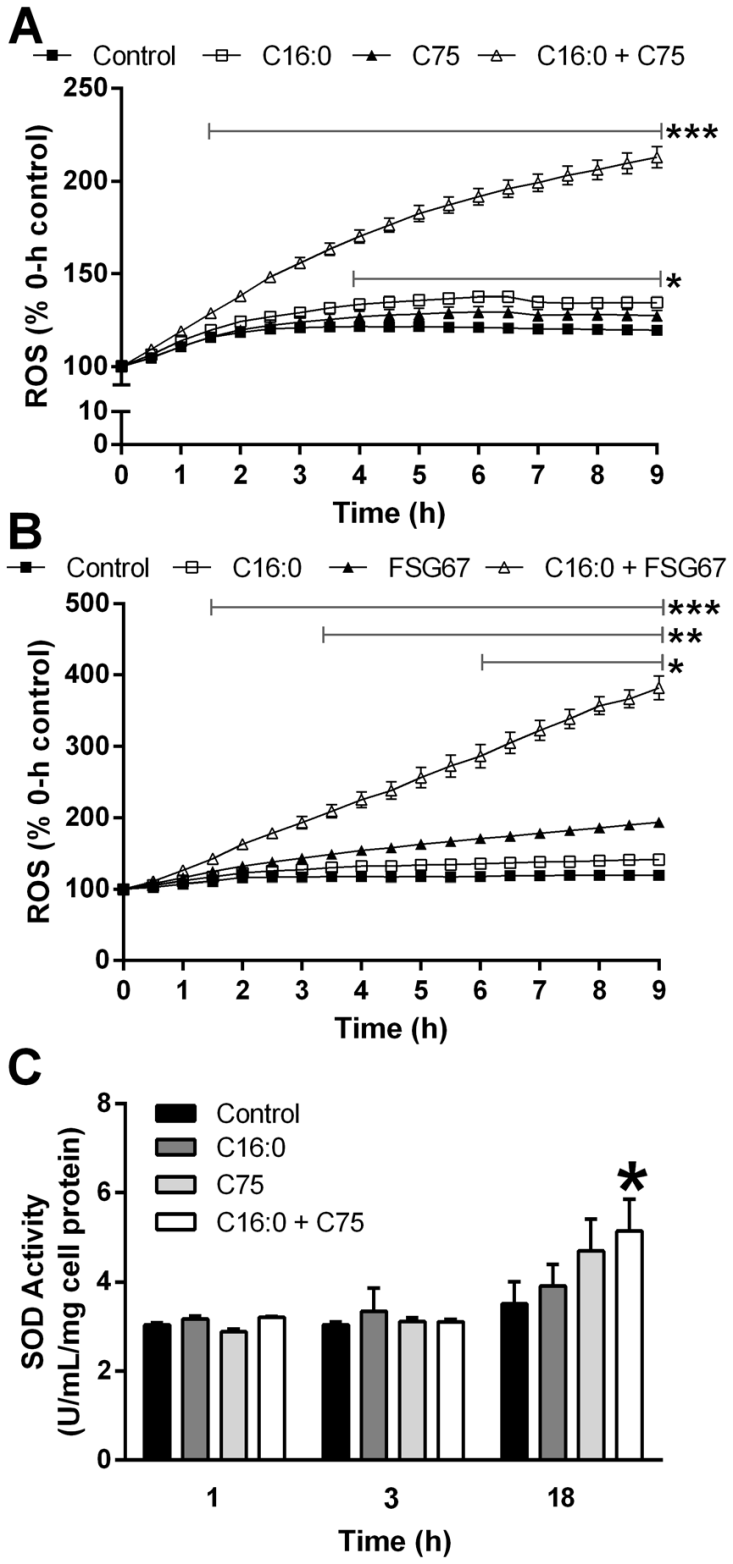
Direct or indirect stimulation of FAOx increases ROS in PHN. Exposure to C16:0 for 9 h increased ROS, an effect potentiated by (A) C75 or (B) FSG67. (C) Acute C75 with or without C16:0 did not affect SOD activity, but 18 h of C75 plus C16:0 increased SOD activity. ROS data were from two independent experiments, five replicates each. SOD activity data were from two experiments, three replicates each. ***, *p*<0.001, C16:0 +C75, or C16:0 + FSG67 versus control; **, *p*<0.01, FSG67 versus control; *, *p*<0.05, C16:0 versus control or SOD data. Data are represented as means ± SEM.

**Figure 7:**
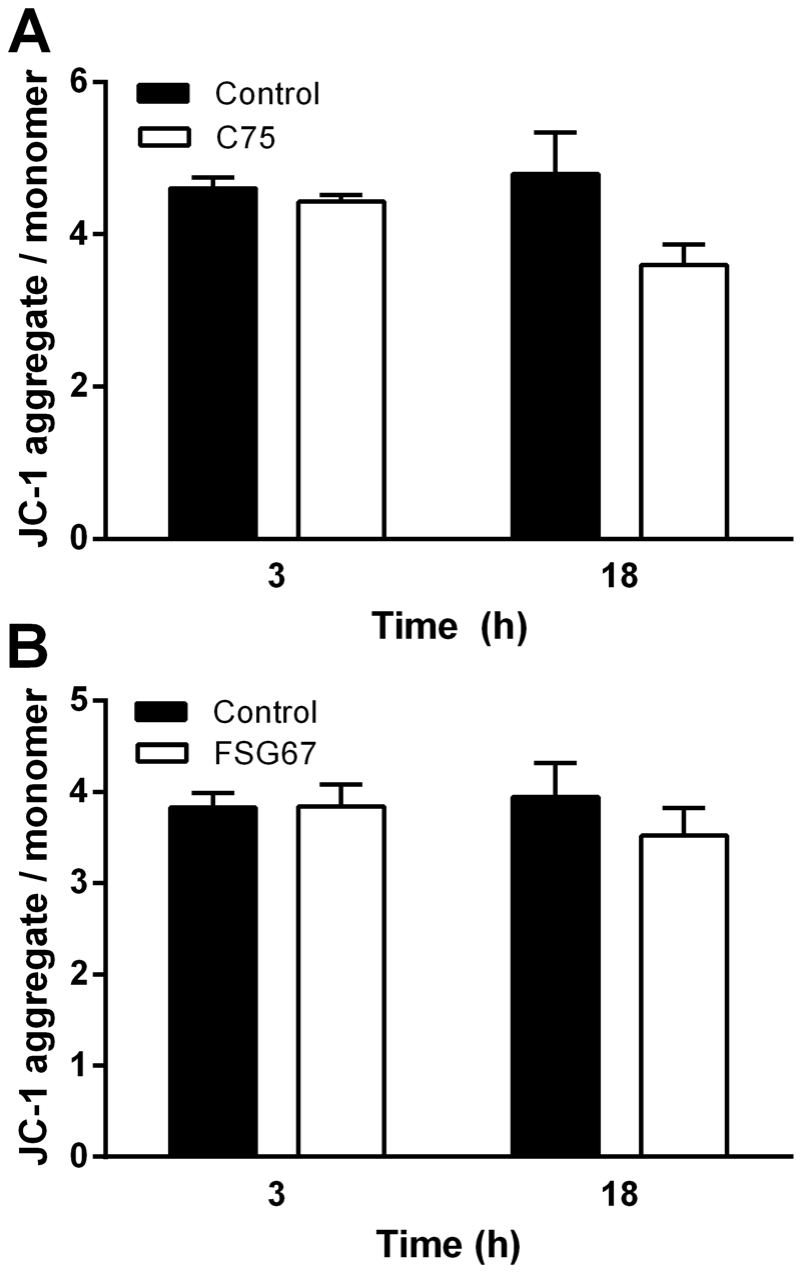
Direct or indirect stimulation of FAOx does not alter mitochondrial membrane potential in PHN. (A) C75 or (B) FSG67 did not alter mitochondrial membrane potential (JC-1 assay). Data are represented as means ± SEM.

C75 and FSG67 both increased FAOx in PHN and neuronal cell lines, so we also examined their effects on neuronal ROS levels and mitochondrial function. Exposing PHN to palmitate (C16:0; FA excess) increased ROS, an effect potentiated by C75 or FSG67 (Fig. 6A, 6B). Responses were similar in PHN cultured with B27 (supplement with antioxidants, linoleate and linolenate) during analysis (data not shown), and in R7HN (S4A, S4B Fig.). ROS can lead to oxidative stress, so we measured antioxidant activity of superoxide dismutase (SOD) in PHN. The presence of either C16:0 or C75 alone did not increase SOD activity; however, C75 for 18 h in the presence of C16:0 increased SOD antioxidant activity (Fig. 6C). Although the increase in ATP levels seen with C75 or FSG67 did not indicate compromised oxidative phosphorylation, increased levels of ROS could impair mitochondria, so we assessed mitochondrial membrane potential in response to C75 or FSG67. Neither C75 nor FSG67 affected mitochondrial membrane potential (Fig. 7A, 7B). These results demonstrate that although the compounds increased neuronal ROS production, they did not compromise mitochondrial health.

### Increasing FAOx shifts metabolic flux away from anabolic processing

**Figure 8:**
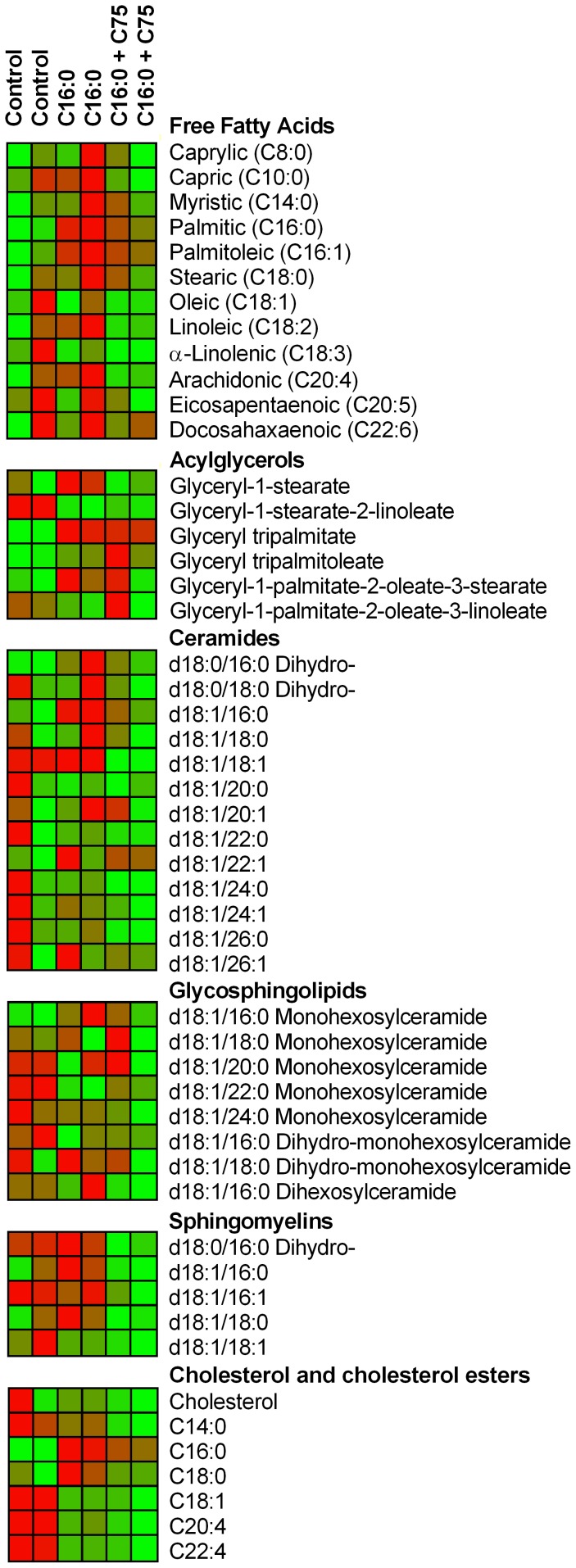
Exposure to palmitate, and increasing FA catabolism with C75, promote lipidomic remodeling in hypothalamic neurons. Targeted lipidomic analysis of PHN treated with vehicle (control), C16:0, or C16:0 + C75 (70 µM) for 3 h. Data are displayed as a heat map of normalized, median-scaled transformed data. Rows represent metabolites and columns correspond to the mean of three pooled replicates (i.e. each treatment had n = 6, 3 per column). Heat maps are calibrated on a twenty-five point color gradient with lowest and highest metabolite levels as green and red, respectively. Data are represented as means ± SEM of normalized, median-scaled data.

**Figure 9:**
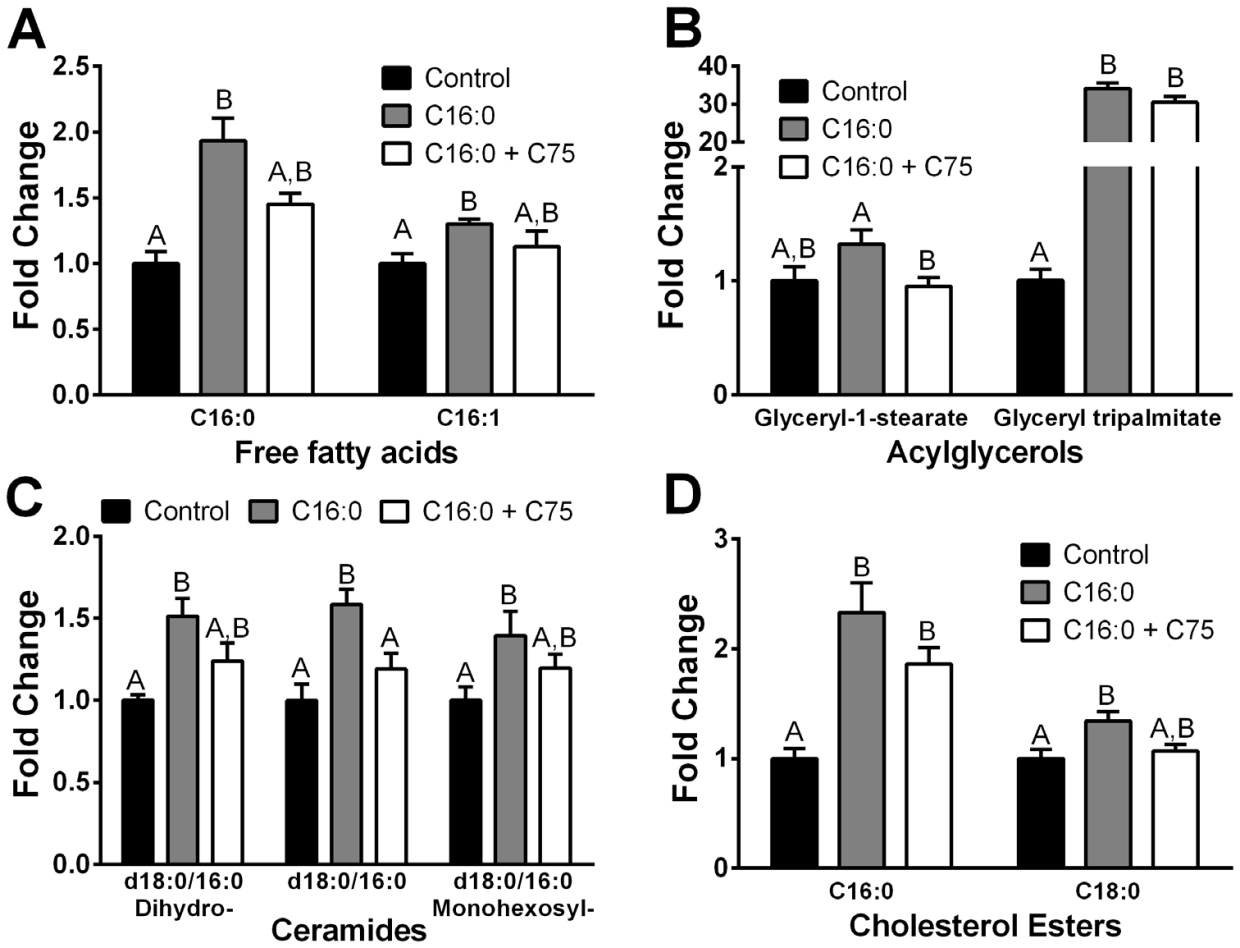
Increasing FA catabolism with C75 mitigates PA-induced production of lipid species. Graphs are representative data of (A) free FAs, (B) acylglycerols, (C) ceramides, and (D) cholesterol esters from Fig. 6. Data are represented as means ± SEM of normalized, median-scaled data. Vehicle controls served as baseline for comparisons within lipid species. Comparisons are made solely within metabolite. Statistics were performed on the log of normalized, median-scaled data. Treatment differences are signified by differing superscripts within metabolite, *p*<0.05.

We evaluated the PHN lipidome in response to excess palmitate, with and without C75 to stimulate FAOx (Fig. 8). Addition of C16:0 increased intracellular free C16:0 and C16:1 (Fig. 9A), an effect prevented by C75 (Fig. 9A). C75 also attenuated C16:0-induced formation of monoacylglycerol (MAG, i.e. glyceryl-1-stearate, Fig. 9B). Excess C16:0 increased glyceryl tripalmitate 35-fold, indicating significant fat levels in PHN (Fig. 9B). C75 did not decrease this triacylglycerol (TAG) synthesis during the timeframe of the experiment. PHN in excess C16:0 had increased ceramides, an outcome reversed with C75 (Fig. 9C). Lastly, although excess C16:0 did not affect overall cholesterol level, it did increase levels of C16:0 and C18:0 cholesterol esters, an effect mitigated with C75 treatment (Fig. 9D). Overall, lipidomics data demonstrated that FA flux shifts away from anabolism with a FAOx stimulator.

**Figure 10:**
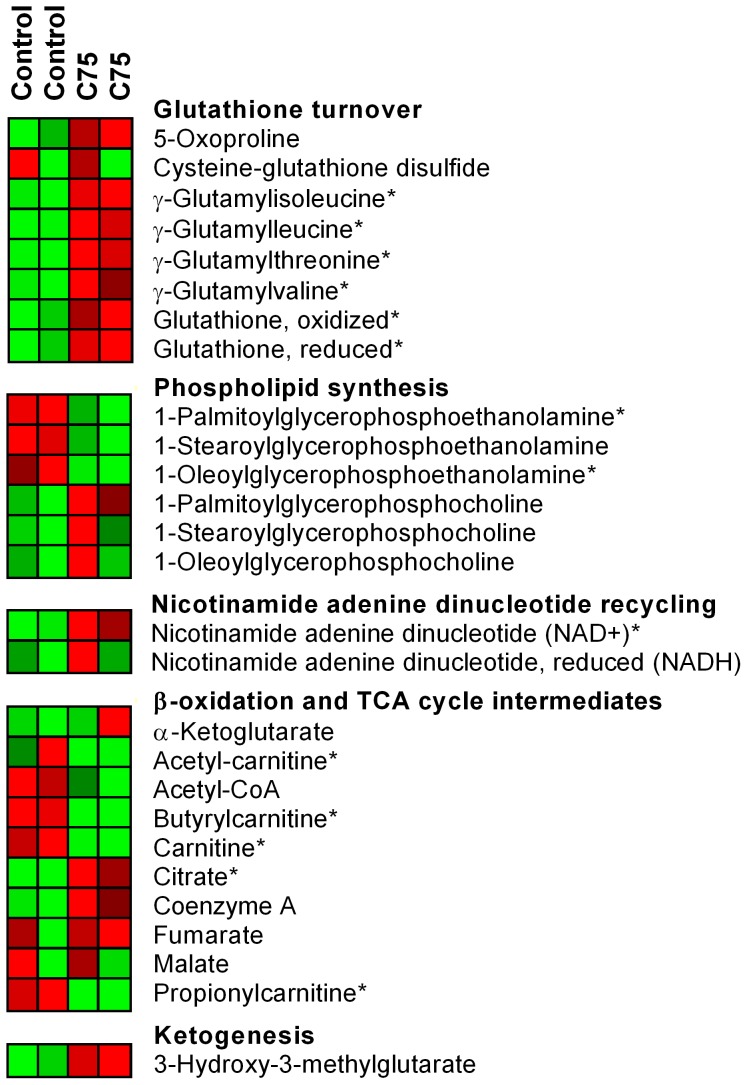
Increasing FA catabolism promotes metabolic remodeling in hypothalamic neurons. Untargeted metabolomics analysis of PHN treated with vehicle (control) or C75 for 18 h, displayed as a heat map of normalized, median-scaled transformed data. Rows represent metabolites and columns correspond to the mean of three pooled replicates (i.e. each treatment had n = 6, 3 per column). Heat maps are calibrated on a twenty-five point color gradient with lowest and highest metabolite levels as green and red, respectively. Comparisons are made solely within metabolite. Statistics were performed on the log of normalized, median-scaled data. Asterisks denote metabolites for which there were significant group differences, *p*<0.05.

We also performed untargeted metabolomics to delineate changes in cellular metabolism in response to increased FAOx in PHN (Fig. 10). Metabolites involved in lipid metabolism were detected. C75 decreased free, unoccupied carnitine, while tending to increase free CoA. This may reflect a shift in the balance of FA being translocated into mitochondria, consistent with the action of C75 as a FAOx stimulator. C75 increased 3-hydroxy-3-methyl-glutarate (3-HMG), product of 3-HMG-CoA hydrolase. This suggests increased steady-state level of 3-HMG-CoA, an intermediate in ketogenesis and an indication of increased acetyl-CoA availability and utilization. Interestingly, levels of 1-palmitoylglycerophosphoethanolamine and 1-oleoylglycerophosphoethanolamine decreased with C75, but corresponding acylglycerophosphocholines were increased. This suggests that C75 may elicit remodeling of cell membranes, not only as regards cholesterol esters (above), but also phospholipid headgroups.

Untargeted metabolomics permitted evaluation of citric acid cycle (CAC) entry molecules and intermediates. C75 remodeled the PHN metabolome in multiple ways to support oxidative metabolism, yet prevent oxidative stress (Fig. 10). Although steady-state levels of acetyl-CoA and acetyl-carnitine decreased, there was a concomitant increase in citrate. Increased β-oxidation with C75 would increase acetyl-CoA supply; however, these data suggest increased acetyl-CoA utilization via upregulated CAC flux, in further support of the hypothesis that mitochondrial function is sustained during C75 treatment, with capacity for increased ATP production. C75 also increased levels of oxidized NAD^+^, needed for glycolysis, the CAC, and to produce the NADH reducing equivalents utilized in the electron transport chain for ATP production.

FAOx-induced increases in ROS could promote oxidative stress. However, C75 increased both the oxidized and reduced forms of glutathione, and increased levels of γ-glutamyl amino acids to regenerate glutathione (Fig. 10). There was a trend for increased 5-oxoproline, a marker of glutathione degradation. Finally, cysteine-glutathione disulfide, an indicator of oxidative stress, did not increase in PHN in response to FAOx with C75. Thus, the metabolomics data show that the increased FAOx is accompanied by metabolic remodeling in PHN that prevents oxidative stress.

### Enhanced FAOx does not increase ER stress in PHN

**Figure 11:**
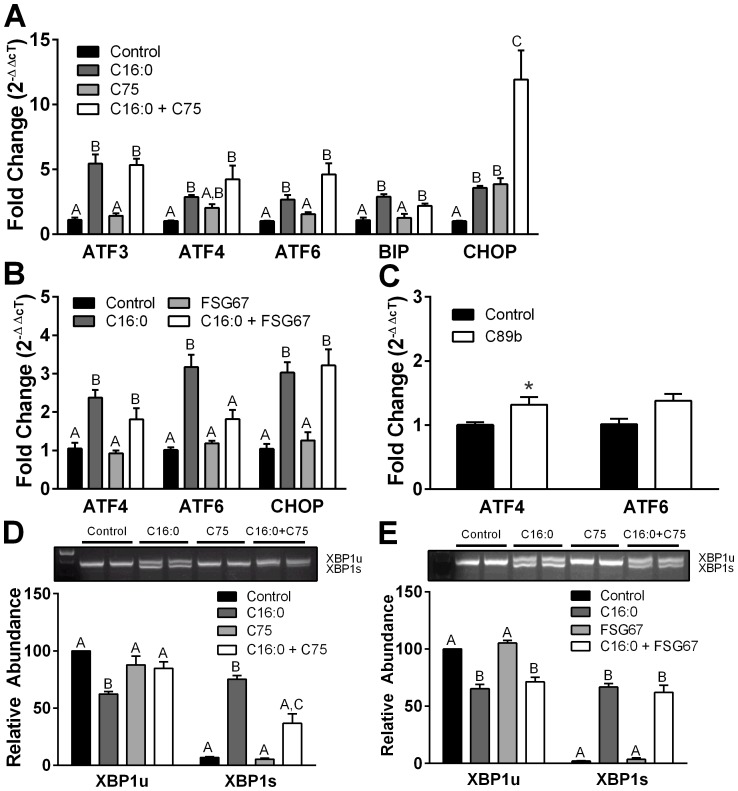
Treatment of hypothalamic neurons with stimulators of FAOx does not evoke a strong UPR. (A) Palmitate (C16:0) in absence or presence of C75 increased ATF3, ATF4, ATF6, and BIP levels. C75 alone had no effect on UPR activation. (B) FSG67 did not elicit UPR in either the absence or presence of C16:0. (C) C89b weakly enhanced ATF4 transcription. (D) C75 or (E) FSG67 did not induce XPB1 splicing; however, C75 prevented C16:0-induced XBP1 splicing. Treatments lasted 18 h. Data were from two experiments, three replicates each. Data are represented as means ± SEM. For C75 or FSG67 effects in PHN, treatment differences are signified by differing superscripts within transcript, *p*<0.05. For C89b data, treatment differences are denoted, * *p*<0.05.

Hypothalamic ER stress from nutrient excess leads to metabolic dysfunction [6]. ER stress induces the UPR to restore ER homeostasis and prevent cell damage. We used thapsigargin (TG) as a validated positive control to deplete ER calcium, to promote ER stress and demonstrate activation of the UPR in PHN. TG upregulated gene expression for UPR markers ATF4 and ATF6 (S5 Fig.), an early event prior to the translation and post-translational activation of these proteins for the UPR [37], [38], and upregulated mRNAs for ATF3 and binding immunoglobulin protein (BiP), gene targets of ATF4 and ATF6, respectively [39], [40]. TG also upregulated mRNA for C/EBP homologous protein (CHOP, pro-apoptotic marker), an outcome downstream of ATF activation, and caused splicing of XBP1 (S5 Fig.). Excess dietary fat leads to lipid accumulation and abnormal intracellular metabolic fluxes that contribute to ER stress [41]. In PHN, excess C16:0 increased expression of ATF4 and ATF6 (Fig. 11A), consistent with results in mHypoE44 cells [26], and increased expressions of gene targets ATF3 (target of ATF4) and BiP (target of ATF6) (Fig. 11A). ROS accumulation can trigger ER damage and protein modification [42]. However, although C75 and FSG67 increased ROS in PHN, neither compound induced ATF6 transcription (Fig. 11A, 11B). Furthermore, C75 did not increase transcriptions of ATF3 or BiP (Fig. 11A). Treatment with the CPT-1 stimulator C89b had minimal effect on ATF transcription (Fig. 11C). C16:0 increased XBP1 splicing in PHN, but neither C75 nor FSG67 stimulated XBP1 processing (Fig. 11D, 11E). Furthermore, C75 partially reversed C16:0-induced XBP1 splicing, a protective response. The data show that these compounds do not induce the UPR, indicating that they do not increase ER stress.

### Increased FAOx prevents C16:0-induced inflammation

**Figure 12:**
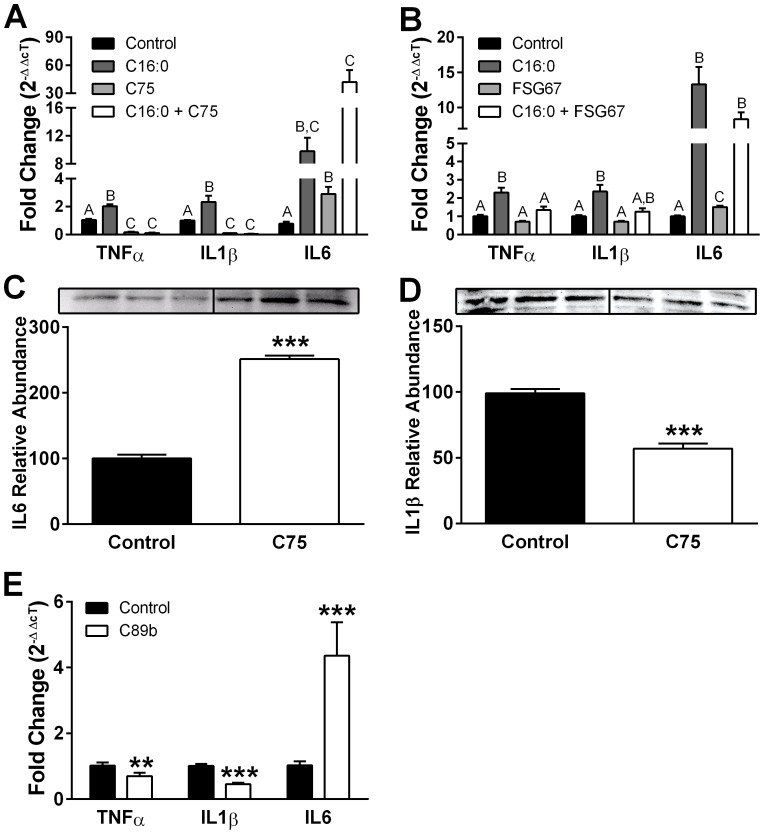
Modifying FA metabolism alters pro-inflammatory cytokine expression in PHN. (A) C75 reversed C16:0-induced mRNA expression for TNFα and IL1β, and potentiated C16:0-induced expression of IL6. (B) FSG67 reversed C16:0-induced mRNA expression for TNFα and IL1β. C75 (C) increased abundance of IL6 protein and (D) decreased IL1β abundance in PHN. Protein data were collected from two independent experiments, three replicates each. Group differences: ***, p<0.001; ** p<0.01. (E) The selective CPT-1 stimulator C89b produced a cytokine mRNA expression pattern like that of C75. For all experiments, treatments were 18 h. Data were collected from two independent experiments, three replicates each. For effects of C75 or FSG67 in PHN, differing superscripts within transcript signify treatment differences, *p*<0.01. For other data: ***, *p*<0.001; ** *p*<0.01. Data are represented as means ± SEM.

High-fat diets can increase hypothalamic saturated acyl-CoA (palmitoyl-CoA) and upregulate inflammatory signaling, impairing insulin responsiveness and appetite control [7], [8]. PHN exposed to C16:0 for 18 h had increased levels of mRNA for TNFα, IL1β, and IL6 cytokines (Fig. 12A, 12B). While TNFα and IL1β elicit pro-inflammatory responses, evidence suggests that hypothalamic IL6 reduces neuronal inflammation and ER stress to restore insulin and leptin signaling and improve energy balance [43]. C16:0 also increased cytokine expression in the R7HN hypothalamic neuronal cell line (S6A Fig.). We hypothesized that increasing FAOx would prevent C16:0-induced inflammation in PHN. Indeed, C75 alone or in the presence of C16:0 suppressed TNFα and IL1β mRNA expression completely in PHN (Fig. 12A). As with C16:0 treatment, C75 treatment increased IL6 mRNA; this increase in IL6 mRNA was potentiated when PHN exposed to C16:0 were treated with C75 (Fig. 12A). Treatment with FSG67 also reversed C16:0-induced TNFα and IL1β mRNA but, unlike C75, did not influence IL6 mRNA expression (Fig. 12B). The hypothalamic neuronal cell line R7HN had similar responses to FAOx stimulators, except that C16:0-induced IL6 expression was not potentiated with C75 (S6A, S6B Fig.). We measured cytokine abundance; C75 increased IL6 and decreased IL1β in PHN (Fig. 12C, 12D), and the CPT-1 stimulator C89b produced a cytokine expression profile similar to that with C75 (Fig. 12C). In all, these results show that FAOx stimulators mitigated the C16:0-induced increase in pro-inflammatory cytokines, and some could enhance an anti-inflammatory cytokine signal.

## Discussion

Recent studies demonstrate that the hypothalamus is susceptible to lipotoxicity and cellular stress that decrease sensitivity to negative feedback signals important for regulating appetite and energy balance, ultimately contributing to the metabolic dysfunction seen in DIO [6]–[8]. Here, we show that manipulating hypothalamic neuronal FA metabolism to remodel the metabolome reverses lipotoxicity induced by excess saturated FA. We treated PHN *in vitro* with FA metabolism modifiers, known to produce hypophagia and weight loss via hypothalamic neuronal mechanisms *in vivo*, in the absence or presence of excess palmitate. We show that both C75 and FSG67 increase FAOx to increase ATP and inactivate AMPK, suggesting that they elicit similar downstream mechanisms. In PHN, both compounds reversed the oxidative stress and indicators of ER stress induced by excess saturated FA. Both compounds also prevented palmitate-mediated induction of the pro-inflammatory cytokines TNFα and IL1β. The PHN lipidome showed signs of increased lipid anabolism during high-fat exposure, that were reversed by enhancing FAOx with C75. C75 also remodeled the metabolome in other biochemical pathways in ways that would support the FA catabolism and energy production, and that would prevent oxidative stress. Overall, shifting hypothalamic neuronal FA metabolism toward catabolism reversed neuronal lipotoxicity and enhanced mitochondrial oxidative phosphorylation to signal energy surplus. These actions may have important implications in hypothalamic regulation of whole-body energy balance.

C75 has been shown to increase FAOx in a variety of cell types using several methodological approaches to assay CPT activity, both *in vivo* and *in vitro*, by stimulating CPT-1a, CPT-1b, and other elements of the CPT shuttle system [16], [17], [44]–[48]. Alternatively, one group indicated that C75 transformed to C75-CoA can inhibit CPT-1 [49]–[51], although they have also shown that C75 stimulates CPT activity [49], consistent with other reports. Antagonizing C75's effects using well-known CPT-1 inhibitors further supports that C75 enhances FAOx [17], [52]. All of the results with C75 in the present studies (effects on AMPK activity, lipidomics and metabolomics, altered CPT-1 gene expression) support that the relevant enzymatic and biological effects of C75 are to stimulate CPT-1 and increase FAOx and energy supply.

FSG67 is a GPAT inhibitor [19], [20]. We considered that FSG67 may also increase FAOx indirectly, by decreasing FA esterification and increasing FA availability to CPT-1. Consistent with this hypothesis, GPAT1 knockout increases hepatic FAOx [53], and hepatic GPAT1 overexpression inhibits hepatic FAOx [54]. Our results show that FSG67, like C75, increases palmitate oxidation and ATP in PHN, and this was verified in hypothalamic neuronal cell lines. It is worth noting that although these and other outcomes are similar for C75 and FSG67 (e.g. hypophagia, weight loss, reversal of palmitate-induced cytokines in PHN), others are not (e.g. expression of cFOS, SREBP1c, and FAS mRNAs in PHN). Thus, we deduce that hypothalamic FAOx and resulting ATP production are the key biological effects sufficient to elicit hypophagia and weight loss. Decreased fat synthesis and ATP usage, though not obligatory, may assist with feeding suppression and weight loss. Thus C75, as a fatty acid synthase inhibitor and a CPT-1 stimulator, may display a more dramatic effect on body weight by affecting these other parameters in addition to the FAOx stimulation.

Both C75 and FSG67 can be used to increase FAOx in hypothalamic neurons, and have been used to decrease food intake and body weight in rodent models. However, the importance of neuronal FAOx in brain metabolism has been debated. The brain as a whole can use FA for energy even under normal conditions [55], but this has been attributed mainly to glial cell types. However, it is important to note that hypothalamic neurons have basal expression of FA oxidative enzymes and transport proteins [56], and equal levels of CPT-1a expression have been demonstrated in cultured neurons versus astrocytes [35]. We have shown that neurons exhibit baseline CPT-1 activity that is inhibited by malonyl-CoA [12] and therefore would be under physiological control by cellular states of energy and fatty acid metabolism. C75 and FSG67 both increased FAOx in PHN. They also led to increased transcription of CPT-1a, which could aid in enhancing FAOx longer-term. Interestingly, levels of CPT1a and CPT1b have been shown to decrease in mouse hypothalamus in response to high level of dietary saturated fat, and the decreased CPT1 was reversed by partial substitution with dietary unsaturated fatty acids [57]. Thus, it may be the decreased availability of saturated FA which occurs with a FAOx stimulator, as we have shown here, that leads to the increase in CPT1a gene expression in PHN. This suggests that maintaining the ability of hypothalamic neurons to oxidize FA is physiologically relevant aspect of their metabolic repertoire.

Enhancing β-oxidation is expected to increase mitochondrial acetyl-CoA availability to the CAC, and thus increase reducing equivalents available for chemiosmosis to produce ATP. Our metabolomics data support this, indicating that C75 increased acetyl-CoA utilization, citrate level, and NAD^+^ recycling in PHN. Conversely, it is known that mitochondrial impairment with nutrient excess decreases catabolic processing and impairs ATP production [58]. FAOx agents may help to restore hypothalamic neuronal mitochondrial function, and thus re-establish appropriate responses to FA sensing even in a setting of overnutrition-induced obesity. As discussed below, we show that in hypothalamic neurons, FAOx agents can decrease levels of potentially harmful lipid species, prevent oxidative stress while permitting fuel oxidation, minimize ER stress, and decrease levels of pro-inflammatory cytokines that arise from over-exposure to saturated FA. These effects should be considered as potential mechanisms by which these FAOx agents can normalize body weight in vivo.

When saturated long-chain FA are in excess, metabolic flux favors synthesis of complex lipids such as ceramides and cholesterol esters, accumulation of which results in lipotoxicity and ER stress, inflammation, and insulin resistance [59], [60]. We confirmed that free C16:0 accumulates in PHN exposed to excess palmitate, and in turn results in increased palmitate-containing ceramides and cholesterol esters. Preventing the buildup of potentially toxic lipids in hypothalamic neurons may be a means to restore anorexigenic signaling in the presence of excess dietary energy. Thus, we explored the possibility that the FAOx stimulator C75 could reverse accumulations of harmful lipids in PHN. We saw a shift in metabolic flux away from anabolic synthesis, with reversal of the C16:0-induced MAG, ceramides, and cholesterol esters. Interestingly, studies have shown that ceramides injected i.c.v. increase food intake, and that the orexigenic hormone ghrelin increases food intake via ceramide production by CPT-1c in the mediobasal hypothalamus [61]. Also, overexpression of CPT-1c in the arcuate nucleus blocks the anorexigenic effect of leptin via increased ceramide synthesis [62]. Ceramide accumulation is toxic, but short-term, site-specific and context-specific increases may be part of normal signaling. One of the effects seen in PHN with C75 was a decrease in expression of the brain-specific, ER-located CPT-1c which would be involved in ceramide production. The potential involvement of decreased ceramide production with C75 in its effects on food intake has not yet been explored.

Although C75 prevented accumulation of ceramides and cholesterol esters with saturated FA palmitate, it did not reverse TAG accumulation in PHN during the time course of study. PHN may have some capacity to store TAG, but when this capacity is overwhelmed, FA may then go to the synthesis of other harmful lipids. Consistent with this notion, levels of ceramides and cholesterol esters increased in PHN with C16:0, but did not increase as much as the TAG. It is important to point out that TAG accumulation may not in itself be lipotoxic, and indeed may be protective, according to recent studies of non-alcoholic fatty liver, whereas FA, especially saturated FA, are harmful in liver and other peripheral tissues [48], [63]. Alternatively, accumulation of hepatic DAG, rather than other lipid species, has been associated with activation of PKCε and hepatic insulin resistance [64], [65]. Investigations of hypothalamic lipotoxicity similarities and dissimilarities to lipotoxicity in peripheral tissues, and specific links to insulin and leptin resistance in hypothalamus have only recently begun.

Oxidative metabolism supports aerobic life, but also produces ROS. Nutrient excess results in sustained and excessive ROS and oxidative stress, leading to mitochondrial dysfunction and impaired ATP production [24], [66]. In PHN, increasing FAOx with C75 under conditions of excess C16:0 did increase ROS; however, the metabolomics data suggest that C75 can prevent oxidative stress. In support of this, mitochondria remained polarized and ATP levels were enhanced with compound treatment, indicating that mitochondrial function was not impaired. The ROS assay we used measures adduct accumulation in cells, leaving the possibility that enhanced ROS clearance could explain the lack of oxidative stress. Indeed, C75 increased glutathione recycling, and addition of C75 increased activity of SOD under the high-fat condition. Interestingly, hypothalamic ROS have been implicated as anorexigenic molecules capable of stimulating pro-opiomelanocortin (POMC) neurons and inhibiting NPY and AgRP neuronal firing to curtail food intake [67], similar to effects of C75 on hypothalamic neuropeptides in vivo [13], [14]. It is possible that moderate ROS production, or perhaps specific ROS species or locations, contribute to the hypophagic effect of C75 and FSG67.

Increased adiposity and insulin resistance are associated with ER protein unfolding. Elements of the UPR mechanism include increased transcription of ATF4 and ATF6, activations of resulting ATF proteins, increased transcription of ATF4 and ATF gene targets, and upregulated XBP1 splicing [37]–[40]. These responses were observed in PHN treated with excess palmitate, thereby confirming UPR activation. In support of our results in PHN and R7HN, mHypoE-44 cells exposed to excess saturated FA show upregulated XBP1 splicing and additional UPR elements including enhanced eIF2α phosphorylation [26]. Exposing PHN to either C75 or FSG67 alone failed to stimulate the UPR, as indexed by no changes in expression of ATF4, ATF6, or splicing of XBP1, and furthermore C75 did not increase ATF3 or BiP, indicating that FA catabolism with these compounds does not induce ER stress. Although the compounds reversed signs of oxidative stress under the high-fat condition, their effects on palmitate-induced signs of ER stress were complex; each compound reversed some but not all ER stress signs. ER stress is indexed by several mediators of the UPR, each of which may be differentially regulated in response to lipotoxicity. It is possible that FSG67 and C75 counteracted different subsets of stimuli for UPR responses, given their different modes of action.

Adiposity and insulin resistance are also associated with inflammation. The gene expression for pro-inflammatory cytokines was increased in PHN exposed to excess palmitate, consistent with data from in vivo studies in DIO [7], and in the R7HN cells. In contrast, other studies using the POMC-positive mouse N43/5 hypothalamic cell line, cultured in non-physiological conditions, indicated no pro-inflammatory response to excess palmitate [68]; discrepancy with the current results may be due to different culture conditions (glucose and oxygen, as well as inclusion of antioxidants), species, or neuropeptide expression profile of the cell lines. Hypothalamic accumulation of palmitoyl-CoA is associated with upregulated inflammatory signaling and local insulin resistance [8]; in response to palmitate we saw increased PHN levels of free C16:0, substrate for palmitoyl-CoA synthesis. We hypothesized that increasing FAOx would prevent C16:0-induced inflammation in PHN. All three FAOx stimulators utilized in these studies suppressed expression and levels of pro-inflammatory cytokines TNFα and IL1β, both under baseline conditions and after inductions from surplus C16:0.

The FAOx compounds do decrease food intake in both DIO models and normal rodents. This suggests a possibility that hypothalamic FAOx may reverse the impaired negative feedback signaling that occurs in the response to high-fat diet, and further speculate that an anti-inflammatory action of FAOx may aid in controlling normal food intake as well. We recognize that non-neuronal glial cells, particularly microglia and astrocytes, are considered to be the main immune-competent cells in CNS, and it seems clear that they are involved in hypothalamic inflammation with lipotoxicity [69]; arcuate nucleus and median eminence neurons showed signs of cell injury (heat-shock protein, autophagosomes, and dysmorphic mitochondria) in rodents on high-fat diet, and both the reactive gliosis and increased cytokine expression that occurred was prominent in these brain sites in just 1–3 days, well before adiposity increased, and again one week later and beyond with chronic high-fat diet [69]. What our data show is that hypothalamic neurons themselves are capable of an inflammatory response to FA excess, and that inducing FAOx in the neurons themselves can reduce this, in a near-absence of astrocytes in vitro. We suggest that neuron-autonomous inflammation signaling may contribute to hypothalamic inflammation responses in vivo. Others reported lack of inflammatory response in one type of hypothalamic cell line in vitro [68], but our experiments using R7HN support that the PHN culture cytokine responses to C16:0, and reversal with FAOx stimulators, were of neuronal origin. The roles, and interplay, of glial cell types and hypothalamic neurons in the course of lipotoxic hypothalamic inflammation in vivo are likely to be complex.

In summary, these studies show that stimulating of hypothalamic neuronal FAOx shifts FA flux away from the synthesis of complex lipids and towards catabolic breakdown to increase ATP supply. Previous studies showed that the appetite-reducing effects of the FAOx compounds studied here are due at least in part to the resulting decreased activity of hypothalamic neuronal AMPK. The current studies point to other potential hypothalamic neuronal mechanisms by which FAOx could restore systemic energy balance in the face of overnutrition. FAOx prevents hypothalamic neuronal lipotoxicity and remodels the metabolome to prevent oxidative stress, ER stress, and inflammation in hypothalamic neurons.

## Methods

### Animal care and use

The Johns Hopkins University Institutional Animal Care and Use Committee approved all protocols as being in accord with National Institutes of Health guidelines for laboratory animal care and use.

### Neuron cultures

Cultured PHN, or N38HN or R7HN (Cellutions Biosystems, Inc.) were utilized. PHN were cultured similar to primary cortical neurons [31]. Conditions for all cultures are in S1 Experimental Procedures.

### Immunocytochemistry

To assess neuron purity, immunocytochemistry was performed on DIV 10 PHN according to S1 Experimental Procedures.

### Treatment preparation

C75 (MW  = 254.2) and FSG67 (MW  = 313.1) were initially dissolved in applicable culture medium at 8.7 mM and 5 mM, respectively (neutralized with NaOH). Thapsigargin (TG) (MW  = 650.8; Sigma-Aldrich) or C89b (MW  = 322.2) were dissolved in DMSO vehicle (cell exposure <0.09%). Compounds were incubated at 37°C and vortexed prior to use. Palmitate was complexed to delipidized BSA. For FAOx, [1-^14^C]-palmitate was incubated with preheated NaOH at 70°C at 1∶1 molar ratio. Sodium salts were diluted and stirred in 37°C culture medium with 1% delipidized BSA for 30 min prior to use. For other experiments palmitate was complexed to BSA according to published methods [26], but DMEM was replaced with applicable culture media; BSA was used in control media. For FAOx, ATP, and AMPK data in PHN, final concentrations for C75 = 100 µM and FSG67  = 160 µM. For other data, in PHN and N38HN, final concentrations for C16:0  = 200 µM, C75 = 70 µM, FSG67  = 160 µM, C89b  = 40 µM, and TG  = 300 nM. For R7HN, final concentrations for C16:0  = 150 µM, C75 = 70 µM, and FSG67  = 160 µM. For ATP data, FSG67- and vehicle-treated PHN were co-treated with 2 µM carnitine.

### Cell viability

Neuronal viability was assessed with membrane-permeable Calcein-AM (Invitrogen) as described [31].

### Oxidative stress markers

Intracellular ROS level was assessed with cell-permeable CM-H_2_DCFDA (Invitrogen) dissolved in DMSO (cell exposure <0.075% DMSO). After removing media, cells in 24-well plates were washed with warm DPBS, loaded with 7.5 µM CM-H_2_DCFDA, and incubated for 45 min. CM-H_2_DCFDA was removed and cells were rinsed in Neurobasal-A medium with zero glucose minus phenol red (custom; Invitrogen), supplemented with 3 mM glucose, 2 mM glutamax-I, 100 units/ml penicillin, and 100 µg/ml streptomycin (Invitrogen). Treatments were then applied in Neurobasal-A wash medium (no B27) and incubated. Fluorescence was measured every 30 min using excitation and emission wavelengths of 492 and 527 nm, respectively. Cytosolic and mitochondrial SOD activity in PHN grown on 12-well plates was quantified with assay kit (Cayman Chemical). Each unit of SOD activity is the amount of enzyme that produces 50% dismutation of superoxide.

### Mitochondrial function

For ATP and mitochondrial membrane potential analyses, cells were grown on 24-well plates. For ATP quantification, cells were lysed on ice in TE buffer (100 mM Tris +4 mM EDTA, pH = 7.5), scraped, boiled, and centrifuged at 18,000×g. ATP in supernatant was measured using ATP Bioluminescence Kit CLS II (Roche). Membrane potential was determined using JC-1 dye (Molecular Probes) as described [31]. Fluorescence data from microplate reader were expressed as ratios of aggregate to monomer.

### Radiolabeled substrate assays

FAox and synthesis was measured as described [12] with modifications cited in S1 Experimental Procedures.

### RNA analysis

Total RNA from PHN or tissue was extracted with TRIzol (Invitrogen) and treated with DNase I (Invitrogen) according to manufacturer's protocol. Real-time quantitative RT-PCR was performed using previous methods [70]. S1 Table shows primer sequences.

### Immunoblotting

Immunoblotting of proteins followed previous methods [31] with modifications as described in S1 Experimental Procedures.

### Metabolomics

For targeted lipidomics, total lipids were extracted from PHN grown on 60-mm dishes by modified Bligh and Dyer procedure [71]. Extracts were analyzed by LC/ESI/MS/MS as described previously [72]. For untargeted metabolomics, metabolites were extracted with methanol from PHN grown on 100 mm dishes. Global metabolomic profiling was performed by Metabolon, Inc. (Durham, NC). Detailed metabolomics methods are in S1 Experimental Procedures.

### Statistical analysis

Data are presented as mean ± SEM. Statistical tests were performed with GraphPad Prism 5.0. Analysis of variance models included effect of treatment, and time when necessary. Significant treatment effects prompted Bonferroni's (ROS) or Tukey's (other data) multiple comparison procedure to identify significant group differences. For metabolomics, statistics were performed on the log of normalized, median-scaled data; however, data are represented as fold-change of median-scaled data. Differences were considered significant at p≥0.05.

## Supporting Information

S1 Fig **Stimulation of FA oxidation increases ATP and inactivates AMPK in N38HN cell line**.
(TIF)

S2 Fig **Expression profile of GPAT isoforms in mouse brain and N38HN.**
(TIF)

S3 Fig **Expressions of SREBP1c and FAS in PHN cultures.**
(TIF)

S4 Fig **Stimulation of FA oxidation increases ROS in R7HN cell line.**
(TIF)

S5 Fig **Thapsigargin elicits UPR in PHN cultures.**
(TIF)

S6 Fig **Modifying FA metabolism alters pro-inflammatory cytokine expression in R7HN.**
(TIF)

S1 Table **Primer sequences used for mRNA analysis.**
(PDF)

S1 Experimental Procedures (PDF)
