# Supplementary figures and images for: Increasing Fatty Acid Oxidation Remodels the Hypothalamic Neurometabolome to Mitigate Stress and Inflammation
Source: PLoS One. 2014 Dec 26;9(12):e115642. doi: 10.1371/journal.pone.0115642 (PMC4277346; doi:10.1371/journal.pone.0115642)

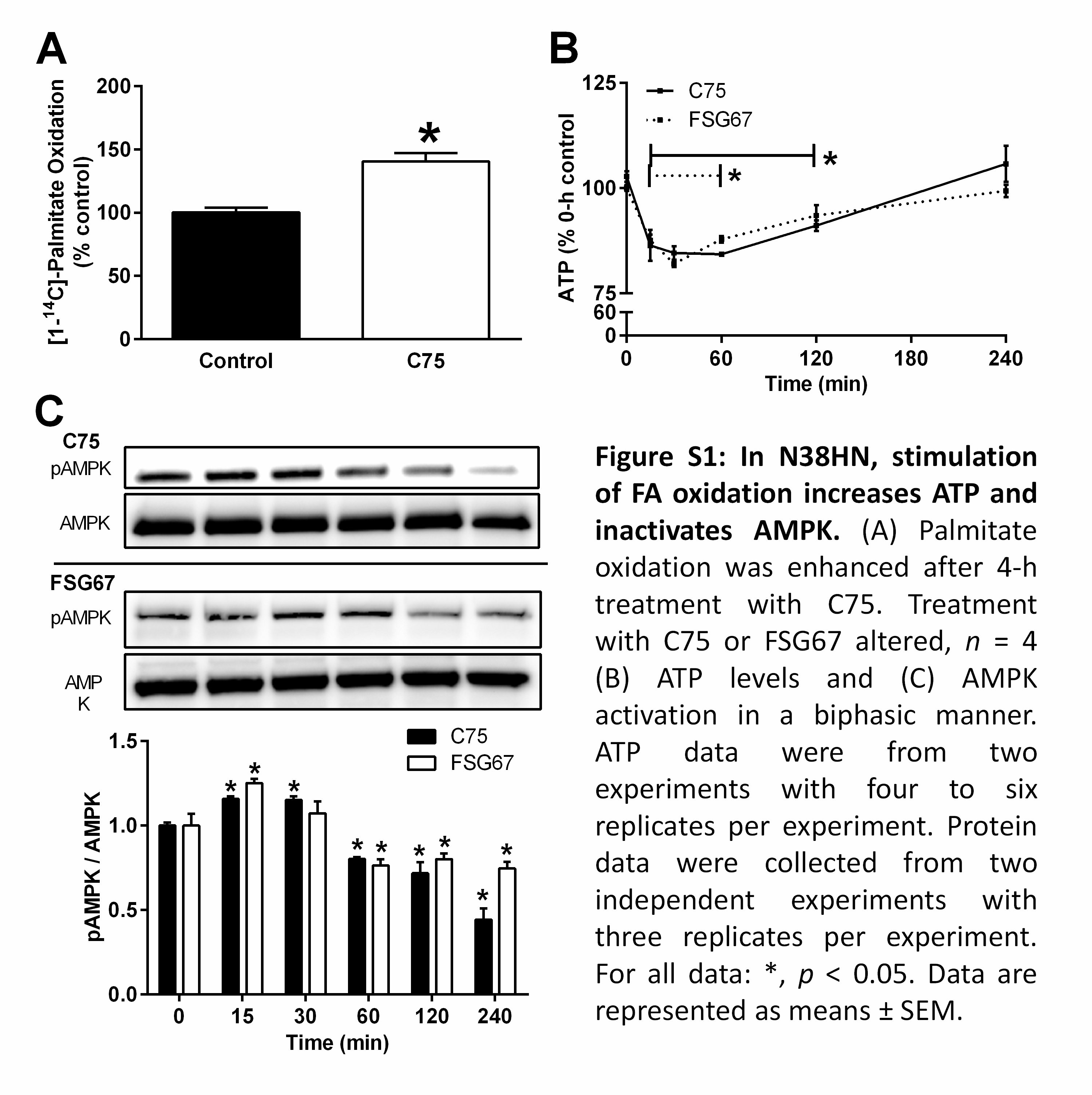

Supplement: S1 Fig — Stimulation of FA oxidation increases ATP and inactivates AMPK in N38HN cell line. (TIF) [file pone.0115642.s001.tif]

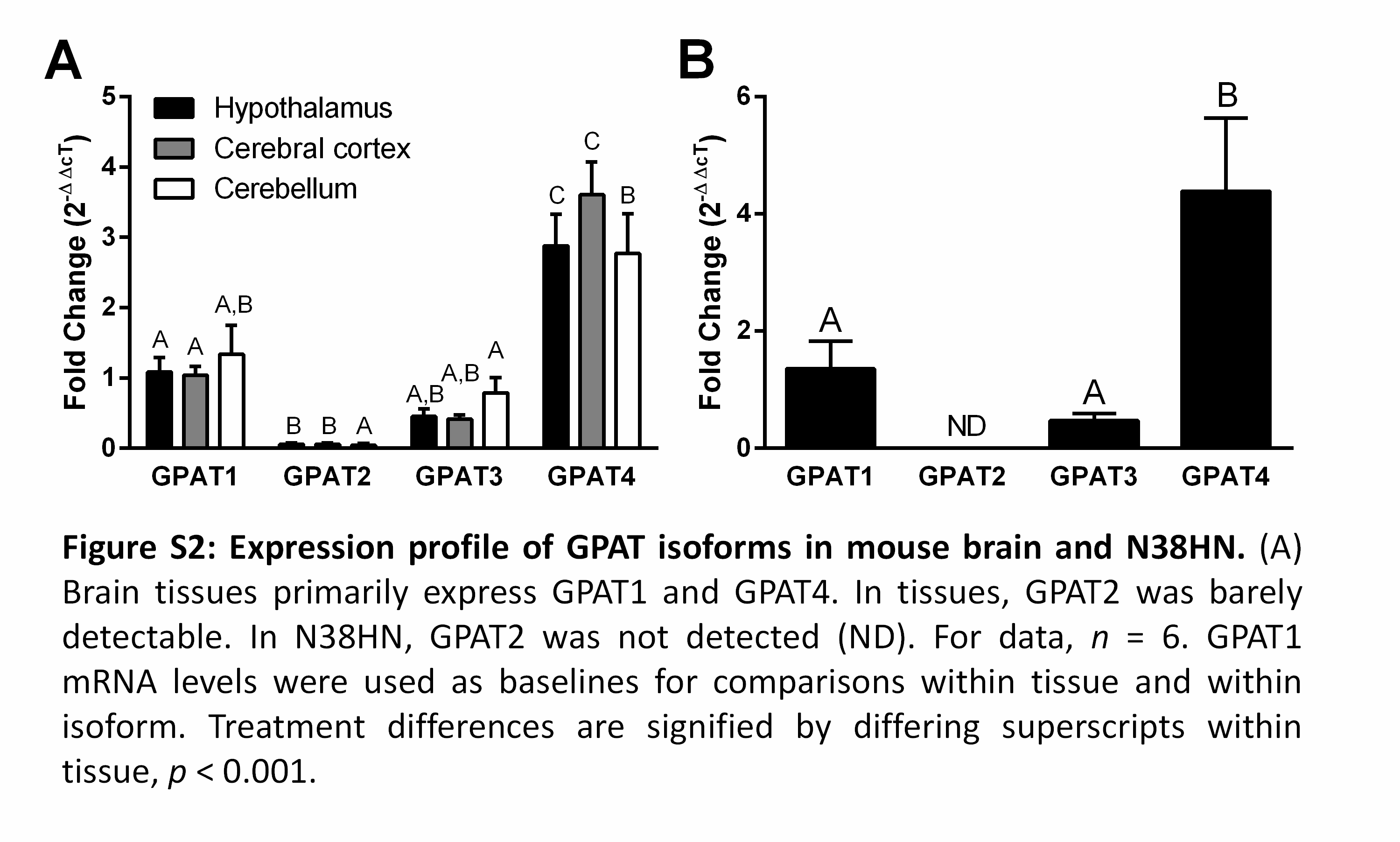

Supplement: S2 Fig — Expression profile of GPAT isoforms in mouse brain and N38HN. (TIF) [file pone.0115642.s002.tif]

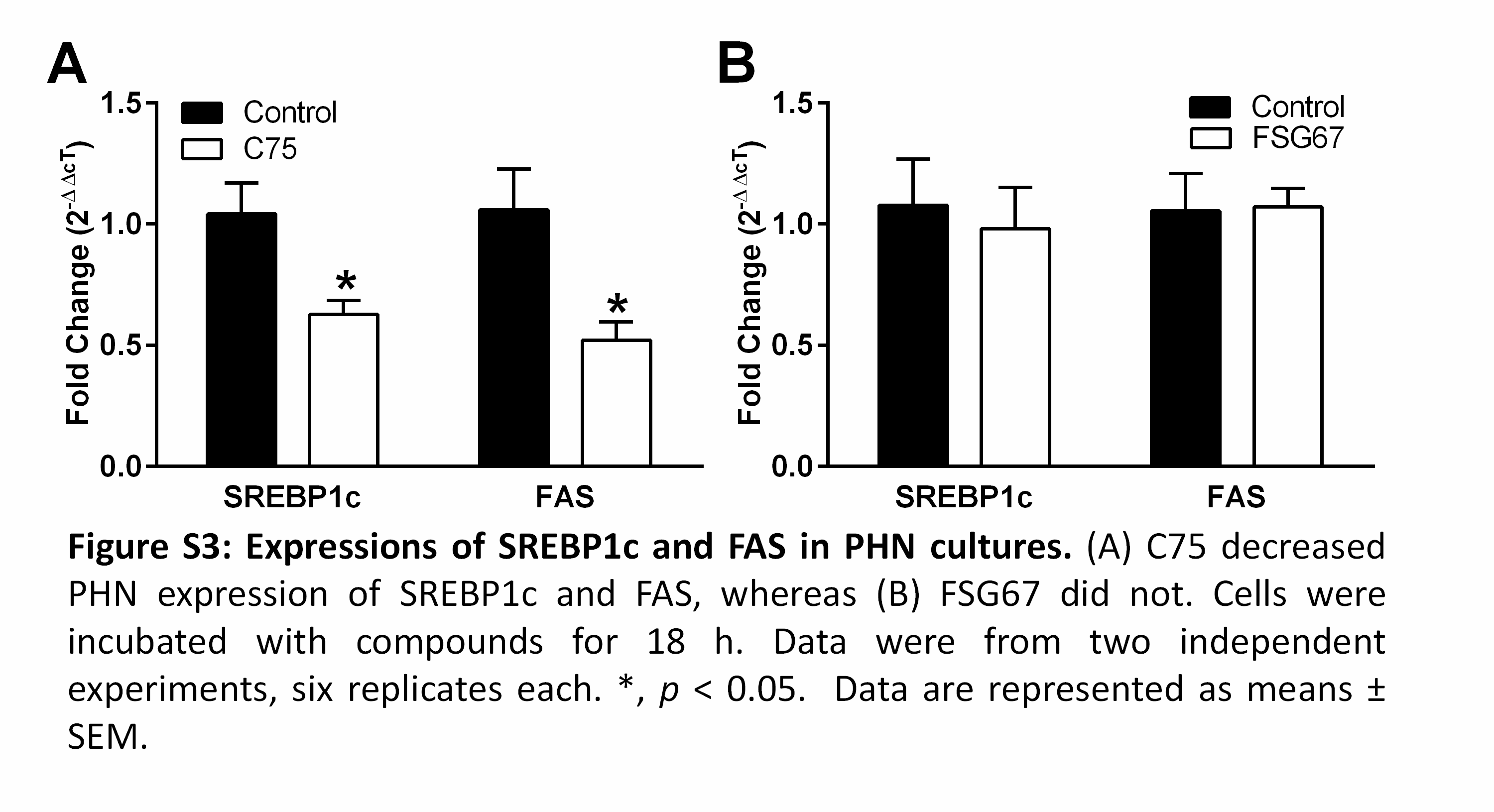

Supplement: S3 Fig — Expressions of SREBP1c and FAS in PHN cultures. (TIF) [file pone.0115642.s003.tif]

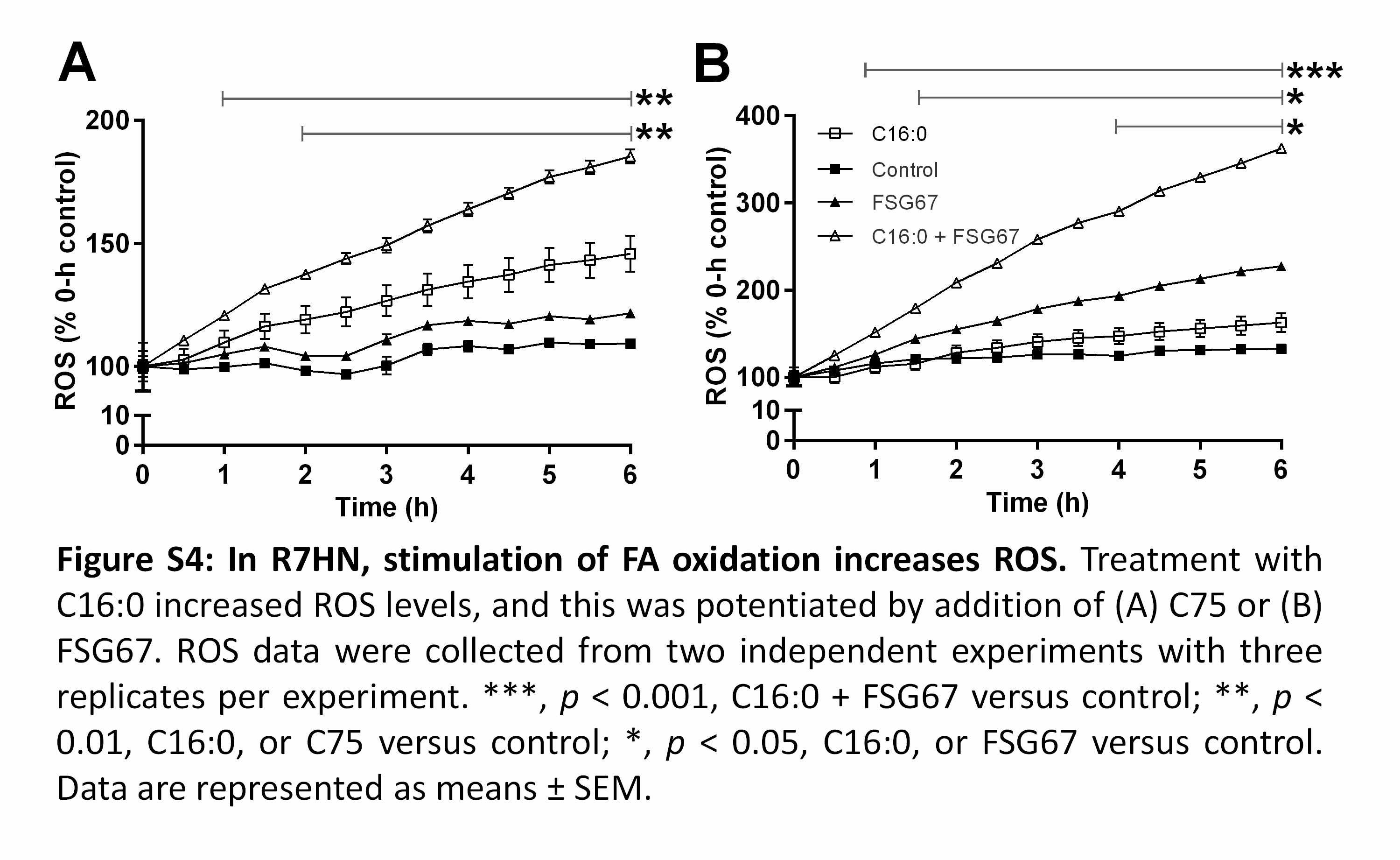

Supplement: S4 Fig — Stimulation of FA oxidation increases ROS in R7HN cell line. (TIF) [file pone.0115642.s004.tif]
