# Supplementary material for: Increasing Fatty Acid Oxidation Remodels the Hypothalamic Neurometabolome to Mitigate Stress and Inflammation
Source: PLoS One. 2014 Dec 26;9(12):e115642. doi: 10.1371/journal.pone.0115642 (PMC4277346; doi:10.1371/journal.pone.0115642)
Supplement: S1 Table — Primer sequences used for mRNA analysis. (PDF) [file pone.0115642.s007.pdf]

Supplemental Table

Table S1: Primer sequences used for mRNA analysis.

Rat	**Forward** (5' → 3')	**Reverse** (5' → 3')	Accession #
cFOS	GTCAACACACAGGACTTTTGCG	CGTGGGGATAAAGTTGGCACT	DQ089699.1
CPT-1a	CGGTTCAAGAATGGCATCATC	TCACACCCACCACCACGAT	NM_031559.2
CPT-1b	CAAACATCACTGCCCAAGCTT	GGCCGCACAGAATCCAAGT	NM_013200.1
CPT-1c	TGCATACCCTGCTCCTGTATC	CCCATCAGCAAGGTCGGT	NM_001034925.2
GPAT1	GTCCAAAGCCATCCAGAAAG	GAAACAAGAGCGGCAGATTC	NM_017274.1
GPAT2	CTCCTGGTTGCAGAGGAGA	AGCAGCTTTGCACTCAGATG	NM_001168529.1
GPAT3	GGAGGATGAAGTGACCCAGA	CCAGTTCTTGAGGCTGCTGT	NM_001025670.1
GPAT4	ATGTTCCTGTTGCTGCCTTT	GGTTCTTCTCCTTGGCTCCT	NM_001047849.1
SREBP1c	GGAGCCATGGATTGCACATT	AGGAAGGCTTCCAGAGAGGA	NM_001276707.1
FAS	TCGACCTGCTGACGTCTATG	TCTTCCCAGGACAAACCAAC	X62888.1
XBP1	AAACAGAGTAGCAGCACAGACTG	TCCTTCTGGGTAGACCTCTGGGAG	NM_001004210.2
ATF4	CCTGACTCTGCTGCTTATATTAC	ACTCCAGGTGGGTCATAAGGTTT	NM_024403.1
ATF6	GGATTTGATGCCTTGGGAGTCAG	ATTTTTTTCTTTGGAGTCAGTCC	NM_001107196.1
CHOP	TTGGGGGCACCTATATCTCA	CTCCTTCATGCGCTGTTTCC	NM_001109986.1
TNFα	TGGGCTCCCTCTCATCAGTT	AGAAGATGATCTGAGTGTGAGG	NM_012675.3
IL1β	CCTGAACTCAACTGTGAAATAGCAG	CAGGTCATTCTCCTCACTGTCG	NM_031512.2
IL6	TGAGAAAAGAGTTGTGCAATGG	GCATCATCGCTGTTCATACAAT	NM_012589.2
ATF3	TCACCTCCTGGGTCACTG	CCGCCTCCTTTTTCTCTC	NM_012912.2
BIP	CCGTAACAATCAAGGTCTACGA	AAGGTGACTTCAATCTGGGGTA	NM_013083.2
L32 (Housekeep	TAAGCGAAACTGGCGGAAAC	CAGGATCTGGCCCTTGAATCT	NM_013226.2
GAPDH (Housekeep	GGTGATGCTGGTGCTGAGT	CAGTCTTCTGAGTGGCAGTG	NM_017008.4
**Mouse**	**Forward** (5' → 3')	**Reverse** (5' → 3')	**Accession #**
GPAT1	GTCCTGCGCTATCATGTCCA	GGATTCCCTGCCTGTGTCTG	NM_008149.3
GPAT2	CATGTTGAAATCCAACCCCCAAACCCAGCA	TGAAACTGTCGGATAAATTGTTCCAGCTTG	NM_001081089.2
GPAT3	GTACATGCCTCCCATGACTAG	GATCCGTTGCCCACGATCATC	NM_172715.3
GPAT4	GGCAGAGGAGCTGGAGTC	TGTTGTGGTACGTAATGATGG	NM_018743.4
18S ribosomal	AGGGTTCGATTCCGGAGAGG	CAACTTTAATATACGCTATTGG	NR_003278.3
