# Supplementary material for: Increasing Fatty Acid Oxidation Remodels the Hypothalamic Neurometabolome to Mitigate Stress and Inflammation
Source: PLoS One. 2014 Dec 26;9(12):e115642. doi: 10.1371/journal.pone.0115642 (PMC4277346; doi:10.1371/journal.pone.0115642)
Supplement: S1 Experimental Procedures — (PDF) [file pone.0115642.s008.pdf]

Supplemental Experimental Procedures

Neuron cultures

Hypothalami were dissected from embryonic (E17) Sprague-Dawley rats (Harlan) and digested in papain (20.1 units/ml) in Earle's balanced salt solution with 10 μg/ml DNAse for 18 min in a 37°C water bath (hypothalamus, cerebral cortex, and cerebellum from 7 adult rats were saved for RNA analysis). Cells were plated onto poly-D-lysine-coated Nunclon plates, dishes, or flasks at 2.0×10^5 cells/cm^2 to 3.0×10^5 cells/cm^2 depending on application. Cells were cultured in Neurobasal-A medium with no glucose (Invitrogen), supplemented with 3 mM glucose, 2% B27 (Invitrogen), 2 mM glutamax-I (Invitrogen), 100 units/ml penicillin, and 100 μg/ml streptomycin (Invitrogen). Cells were maintained at 37°C in a humidified incubator under 5% CO_2, 5% O_2, and 90% N_2. Media glucose levels were measured (Accu-Chek glucometer, Roche) immediately prior to feeding of cells; cells were fed every 3 days by restorative feeding (Kleman et al., 2008) to maintain final glucose at 3 mM. On DIV (day in vitro) 3, cells were treated with 1 μM cytosine arabinoside to inhibit astrocyte proliferation. Assays were performed at DIV 9 or 10. For N38HN or R7HN, neurons were plated on plastic Nunclon culture plates or dishes at 1.6×10^4 cells/cm^2. Cells were cultured in DMEM with 10% FBS, 5 mM glucose, 1% penicillin/streptomycin and maintained at 37°C in a humidified incubator with 5% CO_2, 5% O_2, and 90% N_2 gas mixture. When cells were 90% confluent, FBS was removed, treatments were applied, and assays were performed.

Immunocytochemistry

At DIV 10, media was removed and PHN cultured in 24-well plates were rinsed with PBS and fixed with 4% PFA for 20 min. After DPBS rinse, cells were placed in 10% normal goat serum and 0.1% triton X-100 for 60 min, followed by overnight incubation with primary antibodies in the same solution at 4ºC; co-incubations were rabbit anti-MAP2 (1:1,000; Millipore) plus mouse anti-OX-42 (1:250;

Millipore), or mouse anti-MAP2 (1:200; Millipore) plus rabbit anti-GFAP (1:10; ImmunoStar). The next day, PHN were rinsed with DPBS and incubated with Alexa 488 conjugated goat anti-rabbit, or Alexa 488 anti-mouse secondary antibody, coincubated with Cy3 goat anti-mouse or Cy3 goat anti-rabbit IgG, respectively, for 60 min. Cellular nuclei were counterstained with Hoechst 33342 (1:2000) for 15 min. Immunostained cells were observed with fluorescent microscope (Axiovert 200; Zeiss). Five fields from each well were randomly selected for quantitative analysis. Image-J software was used to count Hoechst stained nuclei, and immunolabeled neurons and glia.

Radiolabeled substrate assays

For FA oxidation, adherent neurons in T25 flasks were treated with compounds for 2 h in Ham's-F10 media. During the last 30 min, 0.5 μCi/ml (20 nmol) of [1-^{14}C]-palmitate (Moravek Biochemicals) complexed to 1% BSA and 2 μM carnitine were added. Flasks were fitted with serum stoppers and plastic center wells containing benzethonium hydroxide. Following incubation, 7% $HClO_4$ was injected into flasks and $^{14}CO_2$ was trapped for 2 h at 37°C. Trapped $^{14}CO_2$ was removed and quantified by liquid scintillation counting. Flask contents were then hydrolyzed with 4 N KOH and neutralized using H_2SO_4. Acid soluble products were extracted using chloroform:methanol (2:1, v:v) and dH_2O and quantified by liquid scintillation counting. Total FA oxidation was obtained by addition of $^{14}CO_2$ and acid soluble products. For measurement of FA synthesis, neurons cultured in 6-well plates were labeled with 100 μM [^{3}H]-acetic acid (PerkinElmer Life Sciences) for an additional 2 h. Lipids were extracted with chloroform/methanol, dried under N_2, and counted using a liquid scintillation counter.

Immunoblotting

Cells grown on 6-well plates were washed with DPBS with 50 mM NaF on ice then scraped in lysis buffer (20 mM Tris-HCl, pH 7.4, 50 mM NaCl, 50 mM NaF, 5 mM $Na_4O_7P_2$, 250 mM sucrose, 1%

Triton X-100 (v/v), 500 mM dithiothreitol, and protease inhibitor (1 pill/10 mL of buffer; Roche)). Lysate was centrifuged at 15,000 × g for 15 min. Supernatant was stored at -80°C for protein quantification and immunoblotting. To ensure equal loading of protein, the BCA kit (Bio-Rad) was used for protein quantification. For protein immunoblotting, lysates were thawed on ice and mixed with 5X sample buffer (312.5 mM Tris-HCl, pH 6.8, 50% glycerol, 10% sodium dodecyl sulfate, 5% β-mercaptoethanol, and trace amounts of bromophenol blue), boiled, and run on Tris-HCl 4-15% linear gradient polyacrylamide gels (Bio-Rad). Following protein transfer from gels to PVDF membranes (Bio-Rad), blots were successively probed with the following antibodies: 1:1,000 antiphosphorylated-AMPKα (Cell Signaling), 1:1500 AMPKα (residues 2-20 of α1 and α2; Covance), 1:200 IL6, 1:200 IL1B (Satna Cruz Biotechnology, Santa Cruz, CA), or 1:1000 CHOP (Thermo Scientific, Rockford, IL) in TBST containing 0.1% Tween-20, 5% protease free BSA and 50 mM NaF. Blots were visualized using SuperSignal chemiluminescence kits (Pierce, Rockford, IL). A 1:10 dilution of the Femto kit was used to detect the pAMPK signal and the undiluted Pico kit was used to detect all other proteins.

Metabolomics

For targeted lipidomics, treated PHN grown on 60 mm dishes were washed with ice-cold DPBS, scraped in HPLC water, then centrifuged at 16,000 × g for 5 min. The supernatant was aspirated, and the pellet was frozen in liquid nitrogen and stored at -80°C until extraction. Total lipids were extracted according to a modified Bligh and Dyer procedure (Haughey et al., 2004). Briefly, each sample was homogenized (Sonic Dismembrator, Fisher Scientific) with one or two 5-second pulses at room temperature in 200 µL of deionized water, followed by addition of 600 µL of methanol containing 53mM ammonium formate with appropriate internal standards, then vortexed. Chloroform (800 µL) was then added, and the mixture vortexed then centrifuged at 1,000 × g for 5 min. The bottom chloroform layer was separated and dried under nitrogen evaporation stream drier. The aqueous

supernatant with protein was dried by vacuum and the protein pellet was re-suspended in deionized water and used for protein quantification by BCA assay. The dried bottom chloroform layer was re-suspended in pure methanol and stored at -80°C until analysis by LC/ESI/MS/MS as described previously (Bandaru et al., 2011; Bandaru et al., 2009). Briefly, chromatographic separations were performed by reverse-phase C18 liquid chromatography columns as a stationary phase and the following mobile elution phases (A) water: methanol: formic acid (59:40: 1, v/v/v) with 5mM ammonium formate) and (B) methanol:formic acid (99: 1, v/v) with 5mM ammonium formate used for the separation of sphingolipids; (C) methanol and 0.1% formic acid, v/v with 5mM ammonium formate, (D) methanol:chloroform (75:25, v/v) and 0.1% formic acid with 5mM ammonium formate used for the separation of acyglcyerols, cholesterol and esters and (E) water and 0.01% formic acid, v/v (F) methanol and 0.01% formic acid, v/v used for the separation of free fatty acids. All solutes were separated by using the gradient elution conditions. Quantitative analyses of ceramides, sphingolipids, DAG, TAG, cholesterol and cholesterol esters were performed by high pressure liquid chromatography (HPLC) coupled to a turbo ion electro spray source of a triple stage quadrupole tandem mass spectrometer API3000 PE Sciex (Applied Biosystems) operated in positive ionization mode, and a 4000Qtrap mass spectrometer (Applied Biosystems) operated in negative ionization mode for the analysis of free fatty acids. Lipid analytes were monitored in multiple reaction monitoring (MRM). Instrument control and data acquisition were performed using Analyst 1.5.1 software.

For untargeted metabolomics, treated PHN grown on 100 mm dishes were washed with ice-cold DPBS then soaked in extraction solvent (80% methanol and 20% ultrapure water with internal standards (D,L-2-fluorophenylglycine, D,L-4-chlorophenylalanine, tridecanoic acid, D6 cholesterol) for exactly 5 min at room temperature. Solvent was collected and stored at -80°C until analysis. The remaining monolayer was scraped in water for protein quantification by Bradford assay. Global

metabolomic profiling was carried out by Metabolon, Inc. (Durham, NC) on three independent instrument platforms: gas chromatography/mass spectrometry (GC/MS), and ultrahigh performance liquid chromatography/tandem mass spectrometry (UHLC/MS/MS2) optimized for basic or acidic species. Detailed descriptions of these platforms, configurations and conditions, data acquisition, and software approaches for data handling, are described (DeHaven et al., 2010; Evans et al., 2009).

Supplemental References

Bandaru, V.V.R., Patel, N., Ewaleifoh, O., and Haughey, N.J. (2011). A Failure to Normalize Biochemical and Metabolic Insults During Morphine Withdrawal Disrupts Synaptic Repair in Mice Transgenic for HIV-gp120. Journal of Neuroimmune Pharmacology 6.

Bandaru, V.V.R., Troncoso, J., Wheeler, D., Pletnikova, O., Wang, J., Conant, K., and Haughey, N.J. (2009). ApoE4 disrupts sterol and sphingolipid metabolism in Alzheimer's but not normal brain. Neurobiology of Aging 30.

DeHaven, C.D., Evans, A.M., Dai, H., and Lawton, K.A. (2010). Organization of GC/MS and LC/MS metabolomics data into chemical libraries. Journal of Cheminformatics 2.

Evans, A.M., DeHaven, C.D., Barrett, T., Mitchell, M., and Milgram, E. (2009). Integrated, Nontargeted Ultrahigh Performance Liquid Chromatography/Electrospray Ionization Tandem Mass Spectrometry Platform for the Identification and Relative Quantification of the Small-Molecule Complement of Biological Systems. Analytical Chemistry 81.

Haughey, N.J., Cutler, R.G., Tamara, A., McArthur, J.C., Vargas, D.L., Pardo, C.A., Turchan, J., Nath, A., and Mattson, M.P. (2004). Perturbation of sphingolipid metabolism and ceramide production in HIV-dementia. Annals of Neurology 55.

Kleman, A.M., Yuan, J.Y., Aja, S., Ronnett, G.V., and Landree, L.E. (2008). Physiological glucose is critical for optimized neuronal viability and AMPK responsiveness in vitro. Journal of Neuroscience Methods 167.
